# Religiosity and gender bias structure social networks

**DOI:** 10.1017/ehs.2024.16

**Published:** 2024-04-08

**Authors:** Erhao Ge, CaiRang DongZhi, Ruth Mace

**Affiliations:** 1 Department of Anthropology, University College London, 14 Taviton Street, University College London, UK; 2 State Key Laboratory of Grassland and Agro-ecosystems, College of Ecology, Lanzhou University, 222 Tianshui South Rd, Lanzhou, Gansu Province 730000, China; 3 IAST, Toulouse School of Economics, Toulouse, Occitanie, 31080, France

**Keywords:** Religion, cooperation, costly signal, social networks, gender bias, C8

## Abstract

The number of studies examining gender differences in the social relationship rewards associated with costly religious practice has been surprisingly low. Here, we use data from 289 residents of an agricultural Tibetan village to assess whether individuals are more inclined to establish supportive relationships with religious individuals in general and to investigate the gender disparities in the relationship between religiosity and personal network characteristics. Our results reveal that participation in religious rituals contributes to the overall development of social support networks. The benefits to personal networks, however, seem to be contingent upon gender. For resource-intensive, infrequent religious rituals such as distant pilgrimages, males seem to benefit slightly more in terms of elevated in-degree values in their personal networks, despite similar levels of investment as females. In contrast, for daily, low-cost religious practices requiring ongoing participation, both genders obtain similar increases in in-degree values through regular engagement. It becomes more challenging for women to increase their status in communities when the effort invested in religious rituals yields smaller rewards compared with the same effort by men, contributing to ongoing gender inequality. These findings highlight the importance of examining the particular characteristics of religious rituals and the gender disparities in the associated rewards.

## Graphical abstract

**Figure:**
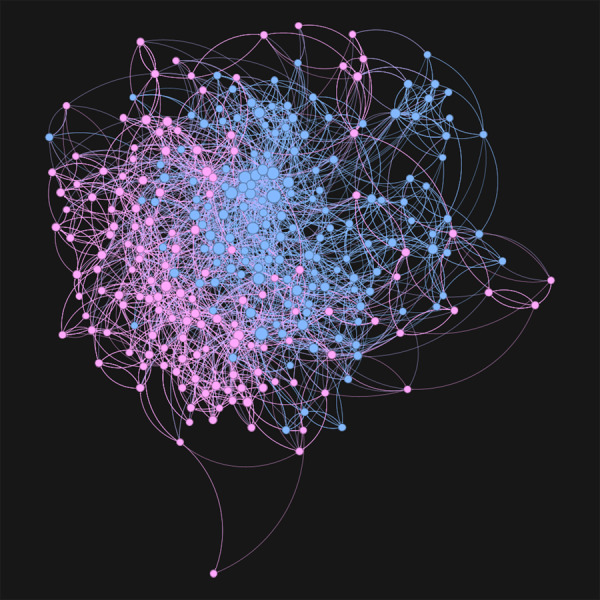

**Social media summary:** Religious practices strengthen social bonds, yet the benefit is swayed by gender.

## Introduction

The engagement of individuals in religious activities or rituals, which often demand significant investments of time, energy and money, or even entail risks to life, presents an evolutionary puzzle. Costly religious practices and rituals have been suggested to facilitate human prosocial tendencies by signalling reputational qualities (Power, 2017a) or even mate qualities to peers (Xygalatas et al., 2022), leading to strengthened trustworthiness among in-group members and promoting cooperation (Henrich, 2009; Norenzayan, 2013; Hall et al., 2015). At the individual level, for example, a study in two villages in South India has shown that residents are more willing to establish trusting relationships with those who engage in regular worship and costlier public religious rituals (Power, 2017b). A longitudinal study of English women indicates that church attendance increases social aid from co-religionists (Shaver et al., 2020), and another study in Bangladesh suggests that women with more religious participation are associated with a greater size of social network (Lynch et al., 2022). At the community level, co-participation in collective religious rituals has been shown to promote social cohesion (Harvey & Lanman, 2014; Singh et al., 2020) and foster denser bonds between individuals (Power, 2018).

Some studies have demonstrated that aggrandising, uncalculating and perhaps infrequent cooperation can help attract new partners and enhance status in the community by broadcasting signals of trustworthiness, garnering more attention and expanding access to additional cooperative partners (Plourde, 2008; Macfarlan et al., 2012; Jordan et al., 2016; Bird et al., 2018). For example, a recent experiment demonstrated that a setup where participants could choose to sacrifice a larger portion of their monetary endowment to play an iterated public goods game in a group with others led to a more efficient grouping of cooperative players (Lang et al., 2022). In real-world scenarios, empirical studies indicated that Meriam marine foragers and Martu hunter–gatherers in Australia extensively share difficult-to-acquire resources as a way to honestly advertise their phenotypic quality as a mate, social ally or another trait important to the observer (Smith et al., 2003), leading to trusting and cooperative partnerships (Bliege Bird & Power, 2015). A recent research using the pilgrimage to Santiago de Compostela as a costly display of commitment suggests that those long-distance pilgrims are perceived as more trustworthy than short-distance pilgrims (Chvaja et al., 2023). Others have found that among the Hadza population, men and younger individuals show a higher willingness to signal their skills as foragers, suggesting that such signalling can be mediated by demographic factors (Stibbard-Hawkes et al., 2023).

However, low-frequency but high-cost performance might play little or even a detrimental role in certain contexts. For example, Power found that individuals are less likely to build a relationship with those who perform grand displays to a large crowd in the annual festival of villages in South India (Power, 2018). Bird et al. showed that hunters who engage in aggrandising strategies failed to gain social support benefits (Bliege Bird & Power, 2015). It might be that observers tend to discount these costly behaviour when they perceive that the actor is eager for direct or indirect financial or social advantage (Raihani & Power, 2021). In a recent experiment, Lang et al. found that high-cost signals were less effective than low-cost signals for assorting cooperators. They speculate that within scenarios demanding significant signalling expenses, free-riders may perceive an opportunity to leverage these costs for exploiting cooperative individuals. Consequently, the efficacy of high-cost signals in accurately identifying cooperators diminishes (Lang et al., 2024). Moderate and persistent prosocial acts might be more efficient in strengthing long-term relationships and social ties (Roos et al., 2013; Bird et al., 2018). A long history of repeated interactions can provide numerous opportunities to personally observe the behaviours of others and assess their reliability, which in turn, can influence social relationships (Granovetter 1973; Oishi et al., 2007; Oishi, 2010; Smith et al., 2016). Sustained prosocial signals, with their frequent and low-cost signs of persistent interest in the relationship, may thus provide more convincing evidence of an individual's value as a long-term friend or collaborator. Moreover, dyadic, routine interactions often play a role in seeking social prominence, a strategy not confined to those overt, dramatic prosocial acts. Utilising longitudinal network data among Tsimane forager–horticulturalist men in Amazonian Bolivia, von Rueden et al. have shown that routine acts of cooperation, such as food-sharing, hunting and fishing, can also be driven by status. Specifically, men of higher status tend to engage more in these everyday cooperative interactions and men of lower status often increase their rank by building such cooperative relationships with higher-status individuals (von Rueden et al., 2019). This may suggest a blurred dichotomy between aggrandising acts motivated by status and the more subtle, less costly behaviours promoting long-term partnerships.

Religious rituals, across various cultures, generally display a broad spectrum of frequency, ranging from daily engagement to once-per-generation events (Whitehouse, 2004), Often, the frequency of a religious ritual is linked to other attributes, such as its level of emotional arousal (Atkinson & Whitehouse, 2011) or the cost to practitioners. One might assume that participation in low-frequency, high-cost religious rituals could be categorised as aggrandising performances which are grand, broadcast, but not consistently affordable signals. Conversely, routine but low-cost religious practices could be seen as moderate performances, characterised as less salient but more persistent signals. The different modes of religious rituals thus may be differentially linked to the establishment of our social structures. Moreover, the preference for or the function of these signals may be differentially influenced by social–environmental factors among participants in real-world settings. For instance, Xygalatas et al. found that individuals with lower social standing participate more frequently and endure greater somatic costs during religious festivals. In contrast, those of higher socio-economic status tend to utilise financial capital to make offerings to the deity (Xygalatas et al., 2021).

Gender is often another key factor of religious participation. It is cross-culturally common that women tend to be more religious than men, although the extent of this gender gap in religiosity varies across cultures (Stark, 2002; Moon et al., 2022; Vardy et al., 2022). Some suggest that this gender differentiation may extend to the relationship between religiosity and cooperation (Ruffle & Sosis, 2007), implying that religious rituals foster cooperation in ways that vary based on gender. In addition, it is generally posited that gender differences exist in the formation, composition and maintenance of social relationships, grounded in sexual selection theory (Emlen & Oring 1977; Benenson, 1990; Geary, 2006; Vigil, 2007; Trivers, 2017). Women tend to form narrow yet cohesive social ties, while men generally have broader but less stable connections (Benenson, 1990, 2019; Sommer 1997). Men are expected to maintain larger networks and form social coalitions (Vigil, 2007; David-Barrett et al., 2015), often navigating status hierarchies (Redhead & von Rueden, 2021) and orienting around mating. Women prefer smaller, more intimate and secure relations (Vigil, 2007; David-Barrett et al., 2015), which may be evolutionarily advantageous for effective child-rearing. However, the empirical findings on the gender-related differences in network structures are mixed, including some conducted in industrialised settings (Turton & Campbell, 2005; Szell & Thurner, 2013; Apicella et al., 2020: 319–329). For instance, von Rueden et al. found that in a society neither matrilineal nor patrilineal, men tend to have larger social networks (von Rueden et al., 2018). Mattison et al. observed that women may have larger social networks in matrilineal contexts (Mattison et al., 2021) but a subsequent comparison between cooperative networks in one matrilineal and one patrilineal community did not reveal clearly divergent network characteristics (Mattison et al., 2023). A study in two Tamil villages in South India provided little evidence of women having smaller and more dyadic networks compared with men (Simpson & Power, 2023). Social ecological factors such as post-marital dispersal patterns (Hruschka et al., 2023; Seabright et al., 2023) and inequalities in material wealth (Redhead et al., 2023) might also mediate the shaping of social networks.

Here, to empirically explore whether and to what extent, participation in grandiose vs. routine religious rituals is associated with social support outcomes and to examine the interplay between gender, religious participation and social networks, we investigated and collected data from a village inhabited by Amdo Tibetan agriculturalists in the northeastern Tibetan plateau in western China. Amdo Tibetan agriculturalists are a deeply religious population with a long history of Tibetan Buddhism. The Amdo Tibetans follow a patrilineal system, and post-marital residence is patrilocal (Zhou et al., 2022). Both polyandry and polygynous marriage were practised in the past, but nowadays, monogamous marriage is predominant (Du & Mace, 2019). Monasteries were centres of learning and authority, integrating the roles of temples, universities, schools, banks and courts (Goldstein, 2010). Religious practice and adherence to Buddhist principles are integral to the daily lives of many Amdo Tibetans, who express their devotion through a range of religious activities, from regularly chanting sutras and prostrating at home to occasional distant pilgrimages undertaken ranging from annually to every few years or even once in a lifetime. Such variance provides an ideal context to investigate the influence of different forms of religious engagement.

We have shown elsewhere that, in this village, individuals perceive engagement in certain religious practices as indicative of various prosocial traits. Specifically, participating in distant pilgrimages enhances the perception of all prosocial characteristics, while daily religious practices are positively linked to perceptions of devoutness but not necessarily to other qualities (CaiRangDongZhi et al., 2023). Here, we draw on measures of religious rituals and network data detailing the supportive relationships within the same village. We first investigate the general association between religious participation and social relationships. We asked the residents to nominate the individuals from whom they ask for assistence when encountering various difficulties. We predict that religious individuals are more likely to be asked for help by their peers. Providers of supports may gain benefits from an increased capacity to receive future support. This can occur either through enhanced prestige by performing costly, helpful behaviours, leading to a preferred status as interaction partners and, consequently, better access to support, or owing to a local cultural emphasis on reciprocity, where recipients of aid may later become sources of support for those who initially helped them. We then examine whether different modes of religious rituals differentially influence individuals’ personal networks. Specifically, we predict that in grand yet infrequent religious participation, such as pilgrimages, males are associated with higher social connections compared with females in this patrilineal, male-privileged setting.

The direction of causation is not always clear in cross-sectional studies. We have shown that religiosity is associated with the enhanced reputation for good characteristics in this village (CaiRangDongZhi et al., 2023) and providing help to others will probably enhance one's reputational status (Power & Ready, 2018). Although we have not specifically studied the relationship between personal reputational standing and social support here, religious participation, prestige and social support are often intertwined. It is hard to untangle whether more religious individuals are more well-respected and therefore have more social connections in the village, or if higher social prestige and more connections motivate religious engagement with peers, or make the engagement of religious rituals much easier. We will not be able to make clear claims about the direction of causality. The association, if present, might be the result of causal directions going both ways, wherein individuals are drawn into rituals through their social connections and, in turn, strengthen their networks by participating in the rituals.

## Methods

### Study area

Sociodemographic data were collected between November and December of, 2019 in an agricultural village in the Amdo Tibetan area, China. The research was approved by the College of Ecology, Lanzhou University. Wealth and land are inherited patrilineally in this village, and the post-marriage pattern is patrilocal. The income of a household often comes from a variety of sources. The majority rely on agriculture, cultivating crops like highland barley, rapeseed, leek, cabbage and potatoes. Additionally, cordyceps, a medicinal fungus, is foraged from remote mountains and sold for substantial income during the slack season (March–May). Some villagers find employment as day labourers or engage in salaried work in town. A few engage in Thangka, a Tibetan Buddhist artwork depicting deities, significantly contributing to their family income.

Residents depend on one another for a variety of different forms of social support. Farmers seek assistance from their peers for planting and harvesting. Neighbourly interactions extend to childcare, errands, food sharing and daily chat about the day's events. Individuals often acquire information about job opportunities and receive financial assistance from their peers. Community members also approach each other for advice on important matters or seek guarantees from their peers. Villagers interact more closely during the annual ritual performances held in June. These religious events, spanning several days, necessitate a high degree of coordination and collaboration amongst villagers or families.

Tibetans primarily practice Buddhism and historically monasteries have been the epicentres of their cultural, political and economic life. Villagers dedicate substantial time to diverse religious activities. They often pray for blessings, such as bountiful harvests or business success, by smouldering highland barley flour and burning cypress. This rituals are routinely conducted by each household on auspicious days or during religious festivals, which have become established rituals that most villagers adhere to at specific times and settings. Moreover, counting prayer beads and reciting sacred texts or sutras are common practices. These acts, often performed voluntarily and privately at home, may occur simultaneously. While not necessarily intended as public displays of devotion, it's common to see individuals carrying their prayer beads and turning them while walking outdoors. As a result, these practices are often inadvertently visible to other villagers. Some residents also perform daily prostrations at home, often maintaining a fixed number of prostrations each day to demonstrate their devotion. While this practice is usually performed alone, the frequent prostrations often polish the wooden floors over time, subtly revealing the resident's devotion to visitors. Villagers also frequently visit local religious sites such as local stupas or monasteries. These visits often occur in small groups during the early morning or evening, with villagers circumambulating a fixed number of times to express their devotion.

Many inhabitants embark on long-distance pilgrimages to various monasteries throughout the Tibetan region as a profound demonstration of their faith. A pilgrimage involves walking to revered sites such as holy mountains, sacred lakes and monasteries, with practitioners prostrating themselves every few steps. The length of these pilgrimages can vary, lasting from a month to over a year, depending on the destination. The financial cost of undertaking a pilgrimage is typically high and increases with the duration and distance of the journey. The longer the pilgrimage, the greater the physical and economic toll, but this is usually seen as correlating with a more profound demonstration of one's devotion. Pilgrimages are often communal and vibrant expressions of faith, with residents travelling in groups that may include family members, friends or fellow villagers. This implies that the act of pilgrimage serves as a signal of devotion that reaches beyond one's immediate family and neighbours. Typically, when someone from the village commences a pilgrimage, the news spreads quickly among the community. It is also not uncommon for some individuals to participate in these distant pilgrimages with their families from a young age.

Over the past several decades, the village has experienced considerable sociodemographic transformations. Governmental interventions, such as the introduction of the people's commune system in the 1950s, the Cultural Revolution in the 1960s, agricultural decollectivisation in the 1970s and the three-child policy and increased educational opportunities in the late 1980s, have significantly impacted the local cooperative behaviour and religious practices. Local monasteries, once the epicentre of Tibetan cultural, political and economic life, were shuttered in 1958 and gradually reopened post-Cultural Revolution in 1976. This reopening, accompanied by a resurgence of previously abandoned religious rituals and practices in the late 1970s and early 1980s (Slobodník, 2004), further shaped the social and religious landscape of the community.

### Data collection

Demographic data were gathered through a household survey. The head of each household was asked about their own, their parents’, siblings’, spouses’ and children's names, gender, age, ethnic group, whether they had ever been a monk, educational level, marital status, birthplace, number of siblings and children (in or out of the household) and occupation, in addition to financial information about the household.

This village has 557 individuals with 398 adults (193 males and 205 females, average age = 43.84 (standard deviation, SD = 15.78)) living in 121 houses among four communities (see Table S1 for more descriptive statistics). The data on social support networks were derived from a survey of all adult inhabitants of this village (289 individuals from 115 households; 147 males and 142 females; 72.6% of all adults). The individuals that are absent from our investigation are largely those who have migrated to a neighbouring town, so residence in the village is sporadic and they infrequently interact with other members of the village. The sample generated here should mostly reflect the complete social network amongst adult village regular inhabitants. We also investigated the religious practices information for these 289 people, including their daily religious activities and pilgrimage information. Consent information was obtained from each survey respondent.

#### Social support networks

In this study, we focused on the complete network comprising 289 interviewees, while excluding ties to six individuals who were not part of our survey owing to a lack of information about their religious practices. One participant was removed from the social support network as nominations were neither made by nor received by this individual. Out of the remaining 288 participants, 287 (comprising 147 males and 140 females) provided nominations, with one participant not nominating anyone but receiving nominations from others. A total of 272 interviewees (142 males and 130 females) received at least one nomination, while 16 participants did not receive any nominations. In this study, respondents were requested to independently identify up to five individuals in response to each question. These specific questions were formulated based on interviews conducted with villagers who possessed significant support networks. The purpose of these questions is to examine various types of support: *emotional support* – characterised by close friendships and engaging in conversations; *behavioural support* – for females, this includes borrowing items, assistance with chores, childcare, and help with family events and for males, it involves aid with farm work and family events; *financial support* – borrowing money and obtaining loans; *guidance support* – seeking advice or discussing important matters; and *guarantee support* – assisting in job search efforts.

These questions were piloted and refined prior to data collection, incorporating feedback from residents. The Supplementary Information provides additional details regarding the questionnaire methodology, as well as descriptive statistics for the networks under examination (Tables S3–S5).

#### Kinship and affinal kinship relatedness

Genealogies were created by linking each person in the census to their mother and father. A person's relationship with living monks was obtained by matching parental IDs between monks and the focal individual. The numeric estimates of relatedness matrices that represent the estimated coefficients of relatedness between the residents and the residents and living monks were calculated from the pedigree data, using the pedigree function in the ‘*kinship2*’ package in R v4.2.0 (R Core Team, 2022).

We determine affinal relatedness using the linking spouse's estimated genetic relatedness (Hughes 1988; Dyble et al., 2018). We identify all spousal and consanguineal relationships in our database via the programme *Descent* (Hagen, 2005). If the relevant couple were married but childless, we manually incorporated spousal links. Affinal relationships were classified into two major categories: spouses of consanguineal relatives and consanguineal relatives of spouses. The affinal relationships are composed of spouses with an affinal relatedness of 1.0; the immediate family of spouses (parents and siblings) and the spouses of siblings, which has an affinal relatedness of 0.5; spouses'consanguineal relatives and consanguineal relatives’ spouses with a cutoff relatedness of 0.25; and spouses’ consanguineal relatives and consanguineal relatives’ spouses with a cutoff relatedness of 0.125. Accordingly, the ‘affinal relatedness’ coefficient is divided into four groups: [0,0.125); [0.125,0.25); [0.25,0.5); and [0.5,1).

#### Religiosity

Most residents are involved in the religious life of the village: 58.5% of villagers reported having participated in at least one distant pilgrimage in the past five years, and 61.2% of villagers reported engaging in daily religious rituals on a regular basis in the past year.

Two distinct variables were used to measure the religiosity of adult residents.

1. *Daily practice.* Individuals are recorded dichotomously as performing daily religious practices regularly if they self-report engaging in at least one of the following activities in the past year: daily visiting a local monastery; performing daily home prostrations; and daily bead counting and sutra recitation. We chose a binary approach over a continuous measure because our primary focus is on the consistent involvement in these practices, rather than on the invariably minimal cost of the practices themselves (see Table S2). Furthermore, we found that accurately quantifying the self-reported frequency of daily practices such as prostrations, bead counting and sutra recitation during interviews is challenging. For instance, some residents struggled to recall the exact frequency of each practice. Others might regularly practice but do not adhere to a fixed frequency, and some only remembered the duration of a focal practice, not an accurate number (see the Supplementary Information for further details).
2. *Pilgrimage score.* We developed a pilgrimage score to quantify the frequency and associated expenses of pilgrimages undertaken by respondents over a five-year period. This metric accounts for a spectrum of pilgrimage activities, ranging from local journeys to monasteries situated in neighbouring towns to extensive pilgrimages to sites located thousands of miles away. Given the geographic distance that pilgrimage endure, the tally of pilgrimage is weighted to account for the relative time, physical and monetary cost entailed in each pilgrimage. This weighting is based on a ranking task conducted with a random sample of 97 residents (50 males and 47 females, average age = 46.16 (SD = 13.14)). Each act is weighted triply from low to high degree on values of 1–5, once for time consumption, once for physical pain and once for monetary cost. We performed a consensus analysis on these rankings with the ‘*AnthroTools*’ package in R v4.2.0 to obtain the combined weighting scores for each category of the pilgrimage (see Table S2). The overall score of pilgrimage for each resident is obtained by the tally where the weighted score of each pilgrimage is multiplied by the frequency with which they conducted the corresponding pilgrimage in the preceding five years (see Supplementary Information for details). For example, an individual who performed three pilgrimages with a weighting score of 15 in the past would gain a pilgrimage score of 45. A pilgrimage with a score of 15 indicates that the pilgrim has travelled thousands of kilometres, whereas a pilgrimage with a score of 4 indicates that the pilgrim has travelled just a few dozen kilometres to visit a local monastery. Given the substantial variation of pilgrimage score (mean value = 12.02 (SD = 18.55)), we standardised it to a mean of 0 and a standard deviation of 1 in the model fitting.

#### Covariates

The number of relatives is a tally of all of the consanguineal relatives that a person has in the village. Economic rank within the village was assessed through a wealth-ranking task conducted by the village head, who possesses in-depth knowledge of each household's economic standing. To facilitate this assessment, we provided the village head with cards featuring the names of each household's primary earner. These cards were subsequently sorted into three distinct categories: wealthy, middle-income and impoverished. To gauge the proximity between households, which serves as a proxy for the physical distance traveled when rendering assistance, we calculated the distance in metres between households using geospatial data from GPS coordinates for longitude and latitude.

### Statistical analyses

#### Sociocentric network analysis

The sociocentric network analyses were performed in the R v4.2.0 using the ‘*igraph*’ package and the ‘*statnet*’ suite of packages. Network visualisation was produced by *Gephi*. We constructed and performed exponential random graph models (ERGMs) on the full social support networks and each type of support network. The full network was a composite of various support networks, permitting overlaps in supportive nominations. In this full network, a dyad was considered to have no tie only if absent in all support networks, and a tie existed if at least one supportive link was present across these networks. Exponential random graph models allow for the incorporation of components at the node (individual), dyad (interpersonal) and network (structural) levels in model (Hunter et al., 2008). Individual (node) characteristics include age, gender, economic rank of the households, pilgrimage score and daily practice; interpersonal (dyad) characteristics encompass gender homophily, community homophily, consanguineous kinship, affinal kinship and the geographic distance between households; and network-level characteristics involve reciprocity and geometrically weighted dyad-wise shared partners in the ERGM for the full network, capturing the tendency for mutual relationships and the heterogeneity in the degree distribution within the social network. We further incorporated ‘In-Degree(0)’ and ‘Out-Degree(0)’ into the network-level characteristics in the ERGMs for each specific supportive network to account for nodes with no incoming or outgoing ties, respectively. These nodes are individuals who have never been nominated to provide a specific type of support (in-degree(0)) or have never nominated others for such support (out-degree(0)). This helps account for the presence of individuals in the village who were either not named by anyone as provider of that particular type of support or did not themselves say that they relied upon anyone within the village for that specific type of support, ensuring a more accurate analysis of the structure in each specific network. However, as such nodes are rare in the full social network, we did not include these two network structure statistics in the corresponding ERGM.

Note that each interviewee responded to the question of who they would turn for assistance when faced with different types of needs. In other words, within each directed tie in the network, the ego represents the individual seeking help, while the alter represents the individual providing help. Our questionnaire does not contain double-sample questions, as we did not ask the interviewees who they have helped. All node-level characteristics in the models, including both socioeconomic and religious variables, are attributes for alters only, that is, the traits of individuals who are nominated as providers of support. The aim here is to evaluate the influence of an alter's attributes on the formation of incoming ties, exploring if an individual's specific characteristics influence the likelihood of them being approached for assistance.

Our social network questionnaire encompassed five types of social support: financial, guidance, and guarantee, along with emotional and behavioural supports. For the first three types, respondents answered only one question each, precluding the possibility of duplicate nominations; hence, there cannot be more than one directed edge between two nodes in the network. However, for emotional and behavioural supports, multiple questions resulted in potential duplicate nominations. For instance, within the behavioural support category, individual *i* might be nominated by individual *j* for both assisting with household chores and child care. However, in these two support categories, the networks are considered unweighted, thereby permitting the existence of multiple directed edges between nodes within these networks. In the process of merging edges from different categories to construct the full network, we analyzed all connections collectively without accounting for their original categories, thereby disregarding network layer interaction. It's noteworthy that overlap may occur between different types of support nominations. For example, individual *i* could be nominated by individual *j* as providing both financial and guidance support. As with the separate networks, the full network is also considered as directed and unweighted, regardless of the strength or frequency of interactions between two nodes.

#### Egocentric network analysis

The egocentric network analysis was performed in R v4.2.0 using the ‘*statnet*’ suite of packages and the ‘*MASS*’ package. To examine the direct social ties of individuals, this analysis focuses on the degree, or the total number of dyadic ties, possessed by an individual. In light of our social network questionnaire where respondents self-reported who they would turn to when in need of assistance, we used in-degree values as a metric of personal network benefits. This measure provides an estimate of the frequency with which a given individual is solicited for aid. We did not utilise out-degree values in our analysis, as these primarily represent the rate at which an individual seeks assistance from others. It is important to note that our focus was on the existence of ties rather than their strength, meaning that we did not differentiate between strong and weak ties based on the frequency of interactions. Each individual's personal social network was constructed by centering on the focal individual, extracting directly adjacent nodes, and incorporating only those edges that originate from direct neighbours and point towards the central node. Within each individual's personal social network, nodes represent individuals who nominated, or sought help from, the central individual, with each edge corresponding to a nomination. As with the full network, personal networks were treated as directed and unweighted, allowing for multiple directed edges between two nodes.

To model the determinants of in-degree values in personal social networks, we employed generalised linear models with a negative binomial distribution, chosen owing to the overdispersed nature of our dependent variable. These models contained the ego's sociodemographic covariates including age cohort, economic rank and the number of siblings and offspring in the village. We compared models incorporating key predictors, including the interaction effect of pilgrimage score and gender, the interaction effect of daily practice and gender, as well as pilgrimage score and daily practice independently. These predictors were examined in separate models. We applied a model selection approach with sets of *a priori*-defined candidate models being compared with determine the best-fitting model according to the lowest second-order Akaike's information criterion.

## Results

### Description

**Figure 1.**
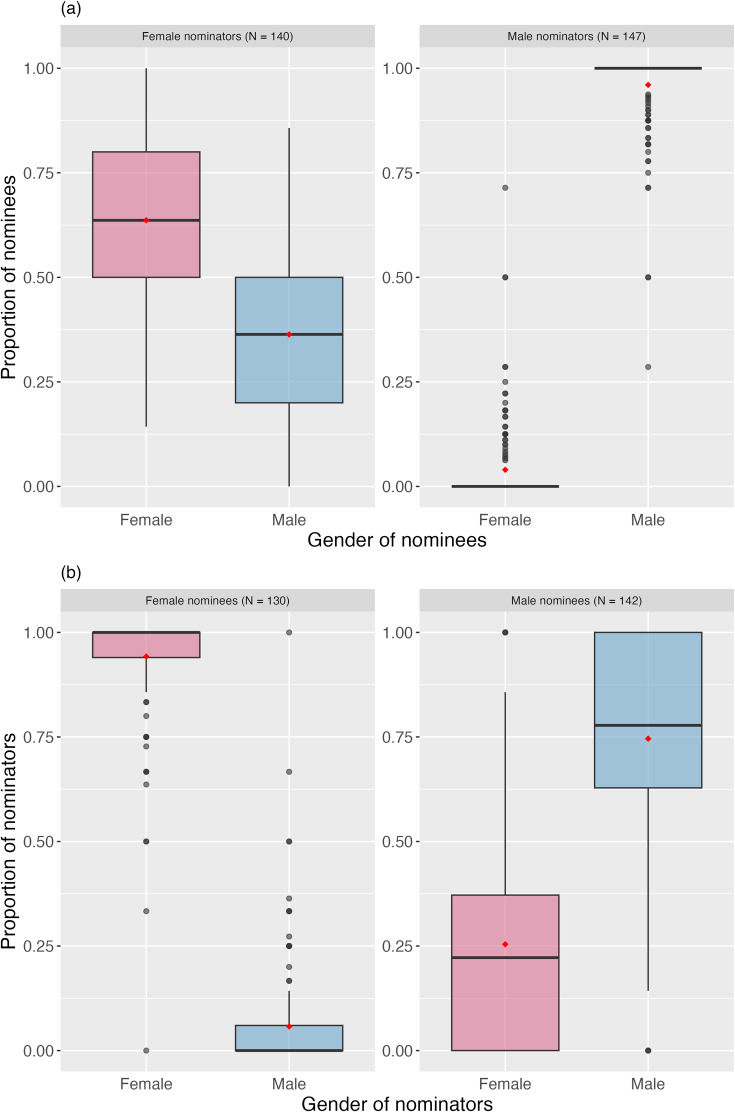
Gender bias in nominations. (a) Gender composition of nominees stratified by male and female nominators. Each boxplot shows the distribution of the proportions of male and female nominees for individual male and female nominators, respectively. (b) Gender composition of nominators stratified by male and female nominees. Each boxplot presents the distribution of the proportions of male and female nominators for individual male and female nominees, respectively. The red diamonds indicate the mean proportion of male/female nominees/nominators. The box represents the interquartile range (IQR), with the central line designating the median. Whiskers extend up to 1.5 times the IQR, while outliers are denoted as dots.

Each nominator (*N* = 287) generated an average of 14.68 (SD = 5.16) names and nominated an average of 10.51 (SD = 3.78) individuals. Each nominee (*N* = 272) received an average of 15.49 (SD = 13.33) nominations from 11.09 (SD = 10.47) individuals. Figure 1(a) shows that for female nominators, an average of 36% (SD = 19%) of nominees are males and 64% (SD = 19%) are females; however for male nominators, an average of 96% (SD = 10%) of nominees are males and only 4% (SD = 10%) of nominees are females, demonstrating that females tend to nominate individuals of both genders, whereas most males tend to nominate predominantly males. Figure 1(b) shows that for female nominees, 94% (SD = 14%) of nominations are made by other women, whereas for male nominees, on average, 75% (SD = 24%) of nominations are from other males and 25% (SD = 24%) are from females (also see Table S4), indicating that females receive nominations mainly from other females, while males receive nominations from both genders.

**Figure 2.**
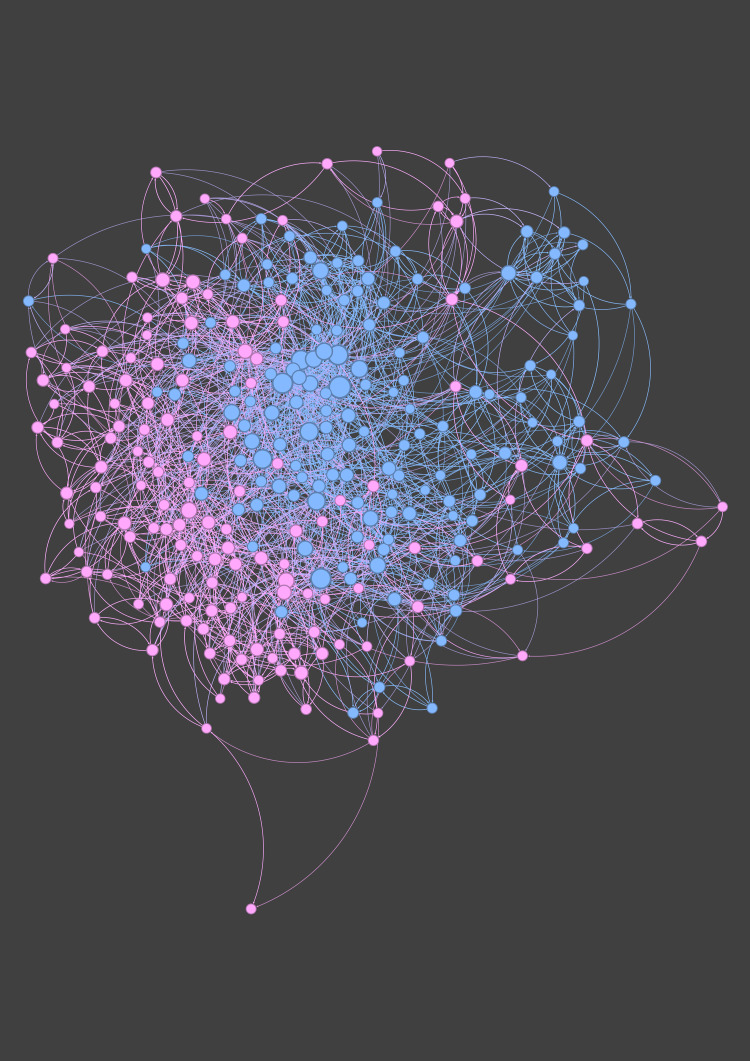
Social support networks among adult residents (nodes = 288, edges = 4214). Each node represents an individual, with colour indicating gender: blue for males and red for females. The size of the nodes corresponds to their in-degree value. The directed edges signify the flow of support, with the arrow pointing from the individual seeking help to the one providing it. The colour of the edge is determined by the gender of the initiator and recipient: where both the initiator and recipient are male it is coloured blue, and where both parties are female it is coloured red. Mixed-gender interactions are represented in grey.The only isolated node is not shown. Networks were produced in *Gephi* using a *Yifan Hu* layout. Also see Figures S2–S6 for visualisation of each specific type of supportive network.

It appears that males take up a larger proportion of individuals with high in-degree values in the overall social support network than females, indicating that males are more likely to be contacted for assistance (Figure 2). Females often rely more on males for certain forms of support, particularly in areas pertaining to Guidance, Financial, and Guarantee support (Figures S2–S7), suggesting a relatively marginal position for females within these social networks. Additionally, we observed a gender disparity in the participation of religious rituals. Daily religious practices seem to be more prevalent among females than males (*χ*^2^ test, *χ*^2^ = 36.206, *p* < 0.001, also see Figure S1). However, when it comes to pilgrimages, the level of engagement did not significantly differ between males and females (Wilcoxon test, *w* = 9660.5, *p* = 0.302). We also found variations in the commitment to daily practices among different age groups (*χ*^2^ test, *χ*^2^ = 60.554, *p* < 0.001), whereas the pilgrimage scores exhibited no significant difference (Kruskal–Wallis test, *χ*^2^ = 4.489, *p* = 0.344). Furthermore, we found no significant correlation between participation in daily religious practice and the economic status of the individual's household (*χ*^2^ test, *χ*^2^ = 0.858, *p* = 0.651). In contrast, there was a significant difference in pilgrimage scores across different economic backgrounds (Kruskal–Wallis test, *χ*^2^ = 9.665, *p* = 0.008). Individuals from wealthier families reported significantly higher pilgrimage scores (Spearman test, *S* = 3387765, *p* = 0.011). These imply that the commitment to daily practice might be more related to the time expenditure, whereas engagement in pilgrimages seems to be more influenced by the availability of resources.

### Religiosity is associated with the development of social support networks

The results from our ERGM fitting the full supportive network (Table 1, also see Table S6 for model selection) show that the standardised pilgrimage score variable significantly influences the formation of supportive ties. Each increase of one standard deviation in the pilgrimage score raises the log-odds of forming a supportive relationship by 0.127, suggesting a 13.6% increase in the odds of forming supportive ties. Additionally, individuals who regularly engage in daily religious practice have a 0.158 higher log-odds of establishing supportive ties compared with those who do not, corresponding to a 17.1% increased odds. Individuals with higher pilgrimage scores and regular participation in daily religious practices tend to be sought after more frequently for support by other residents.

**Table 1.** Exponential random graph model predicting the log-odds of a tie in the full adult support network

	Full Model			
Variables	Estimates	95% CI	OR	*p*-Value
Edges	−4.532	−4.829, −4.235	0.011	<0.001
*Religiosity*				
Pilgrimage score	0.127	0.093, 0.162	1.136	<0.001
Daily practice (yes; ref: no)	0.158	0.037, 0.279	1.171	0.010
*Covariates*				
Age (unit: year)	0.012	0.008, 0.016	1.012	<0.001
Gender (male; ref: female)	1.002	0.871, 1.13	2.723	<0.001
Economic rank (Low; ref: high)	0.012	−0.110, 0.135	1.013	0.8
Economic rank (middle; ref: high)	−0.135	−0.260, −0.010	0.874	0.034
Same community	0.920	0.836, 1.004	2.508	<0.001
Same gender	1.004	0.900, 1.109	2.730	<0.001
Relatedness	1.076	0.602, 1.550	2.934	<0.001
Affinal relatedness	0.047	−0.015, 0.108	1.048	0.13
Geographic distance (unit: metres)	−0.0036	−0.0041, −0.0031	0.996	<0.001
*Structure terms*				
Reciprocity	3.243	3.056, 3.430	25.610	<0.001
Geometrically weighted dyad-wise shared partners (*α* = 0.5)	−0.127	−0.142, −0.111	0.881	<0.001

CI, Confidence interval; OR, odds ratio.

Other factors also significantly influence social relationship structures. Individuals are more likely to seek support from men than women (*β* = 1.002, 95% confidence interval (CI) 0.871–1.130, odds ratio (OR) = 2.723, *p* < 0.001), and are more likely to connect with consanguineous kin (*β* = 1.076, 95% CI 0.602–1.550, OR = 2.934, *p* < 0.001). Socioeconomic status does not seem to influence requests for assistance in general, as there is no significant difference observed between low and high economic ranks (*β* = 0.012, 95% CI −0.110–0.135, OR = 1.013, *p* = 0.8). Community and gender homophily significantly structure the formation of supportive relationships. Individuals are 2.508 times more likely to seek support from those within the same community (*β* = 0.920, 95% CI 0.836–1.004, OR = 2.508, *p* < 0.001), and 2.730 times more likely to seek assistance from those of the same gender (*β* = 1.004, 95% CI 0.900–1.109, OR = 2.730, *p* < 0.001). Reciprocity emerges as the strongest predictor in the network, indicating a pattern of mutual nominations. Individuals are considerably more likely to nominate someone as an assistance provider who is also nominating them simultaneously (*β* = 3.243, 95% CI 3.056–3.430, OR = 25.610, *p* < 0.001).

We then explored the association between religious actions and specific forms of support. The ERGMs were run with networks for each of the five types of support: emotional, behavioural, guidance, financial and guarantee support. The estimates for the religious variables are shown in Table 2, and the full model results are provided in Table S12 (also refer to Tables S7–S11 for model selection for the best-fitted ERGM for each individual social network). It appears that the global pattern of the pilgrimage score aligns with the full network results, with the standardised pilgrimage score significantly promoting the formation of supportive ties across all five types of support. However, the engagement in daily religious practices does not always show significant association across all five types. Specifically, these practices do not notably increase the probability of receiving requests for financial, guarantee or emotional support. They do, however, correlate with an increased probability of providing behavioural and guidance support to others. This suggests that residents might evaluate their peers’ capacity to provide specific types of assistance, which may sometimes necessitate a certain level of economic resources and social standing from those being solicited for help. In addition, gender preferences exist in four out of the five support types, with emotional support being the exception (see Table S12). Compared with women, men are more likely to be sought after by other individuals for behavioural, guidance, financial and guarantee support. Individuals with higher economic status are more likely to be sought for guidance, financial and guarantee support. In contrast, personal economic status does not significantly influence the likelihood of an individual being sought for emotional and behavioural support.

**Table 2.** Exponential random graph model estimates of religious variables in different types of support. Significant effects are in bold.

	Emotional			Behavioural			Guidance			Financial			Guarantee		
	Est	95% CI	*p*	Est	95% CI	*p*	Est	95% CI	*p*	Est	95% CI	*p*	Est	95% CI	*p*
*Religiosity*															
Pilgrimage score	**0.068**	**0.007, 0.129**	**0.028**	**0.159**	**0.110, 0.207**	**<0.001**	**0.156**	**0.102, 0.210**	**<0.001**	**0.083**	**0.005, 0.160**	**0.037**	**0.143**	**0.076, 0.210**	**<0.001**
Daily practice (yes; ref: no)	0.070	−0.089, 0.230	0.4	**0.222**	**0.079, 0.365**	**0.002**	**0.248**	**0.051, 0.445**	**0.014**	0.087	−0.166, 0.339	0.5	0.123	−0.123, 0.369	0.3

Est: Estimated parameter.

### Gender-dependent influence of religiosity on social support in personal networks

In general, males tend to receive more requests for help than females (see Figure S7). As illustrated by the descriptive data (Figure S10a), male practitioners with higher pilgrimage scores seem to correlate with higher in-degree values, implying a more frequent occurrence of being asked for assistance. In contrast, for female practitioners, higher pilgrimage scores do not necessarily lead to an increased frequency of being sought for help. Investment in pilgrimage appears to be more closely associated with males’ social connections than with females’. The pathway to achieving high social support through involvement in pilgrimage seems to be more limited for females than for males. Specifically, female pilgrimage scores show minimal correlation with in-degree values across behavioural, emotional, financial and guidance support types within their personal networks (see Figure S8). Moreover, regular involvement in daily practices appears to be associated with an increase in the in-degree value for both genders (see Figures S10b and S9 for a detailed correlation between daily practice and the in-degree value across each specific types of personal networks).

**Figure 3.**
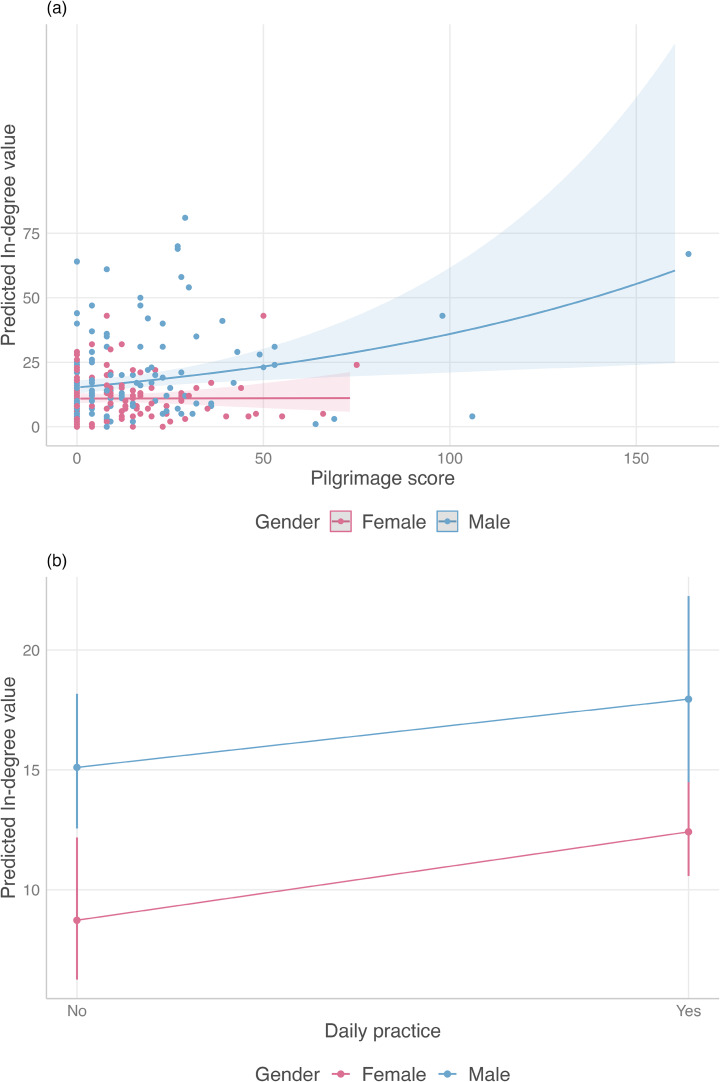
Predicted in-degree values as a function of interaction between gender and religiosity. (a) The *x*-axis represents an unstandardised pilgrimage score, ranging from low to high. The scatterplot displays the observed pilgrimage scores and in-degree values of personal networks. The lines represent the predicted in-degree values for any given pilgrimage scores, based on the fitted model that includes the interaction between gender and pilgrimage score (Model 4 in Table S13). The shaded area indicates the 95% confidence intervals. (b) The *x*-axis illustrates the presence or absence of regular engagement in daily practices, and the *y*-axis represents the predicted in-degree values based on the fitted model that includes the interaction between gender and daily practice (Model 3 in Table S13). The error bars represent 95% confidence intervals. Detailed model parameter estimations can be found in the Supplementary Material, Table S13.

After adjusting for sociodemographic factors in our generalised linear models, we found significant associations among religiosity, gender and personal network benefits. Across all models, gender consistently demonstrated a significant influence, with males exhibiting a higher in-degree value than females (as detailed in Table S13). The model that includes an interaction between gender and pilgrimage score as predictors reveals a trend towards a marginally significant effect (see Figure 3a, and Model 4 in Table S13). This implies that the influence of pilgrimage scores on in-degree value may vary by gender, aligning with the descriptive pattern observed: males with higher pilgrimage scores are associated with higher in-degree values, a correlation that is less apparent for females. In a separate model examining the interaction between gender and daily practice, we did not find a significant interactive effect, as illustrated in Figure 3b (also see Model 3 in Table S13 for model parameter estimates). This suggests that the association between daily practice and in-degree value does not differ substantially between genders, hinting at a potential for both genders to be associated with social rewards through regular participation in daily religious rituals. The inclusion of interactive terms in the models does not appear to significantly enhance explanatory power compared with the model without these terms (see Table S14 for further details). In the latter, pilgrimage score and daily practice are significant predictors of higher in-degree values. These results highlight the role of religious participation in developing and structuring personal networks, and potentially imply an interplay between gender and religious practice in determining network benefits.

## Discussion

Overall, these results provide evidence that residents’ relationships in this indigenous village are strongly influenced by their religious behaviours. Residents are more likely to establish supportive bonds with those who engage in both ordinary, daily religious practices and grand, infrequent pilgrimages. Pilgrimage is linked to a greater number of personal network connections, with a trend of men benefiting slightly more. Males and females who regularly engage in daily practices tend to have a similarly increased number of social connections.

The gender preference for religious practices may be somewhat attributed to gendered division of labour. Owing to constraints of time and financial resources, women may find it challenging to participate in extensive religious performances, such as pilgrimages, and are more likely to engage into those routine practices in the snippets of time afforded by their schedules. Social norms may also prevent women from attending some public rituals. We observed that only men are permitted to perform certain rituals or enter a specific religious venue in the process of investigation. Moreover, the gendered division of labour may also shape the social network dynamics within the community, with men tending to engage in more labour outside of the household and having more mobility or opportunities to participate in larger groups (von Rueden et al., 2018). This preference is more likely for women if they have more discretionary time. For example, some suggest that perimenopausal women in some societies have more opportunities to have a greater number of cooperation partners owing in part to fewer parenting responsibilities (Olesen 1987). The division of labour tends to steer women towards more intra-household work. Women's need for intimate and bilateral relationships may be greatly increased because such relationships are more helpful to women in childcare and other domestic labour. Or it could also be that women are more likely to form intimate relationships with those who help them with childcare and domestic labour, although it may sometimes limit women's mobility and constrain women's socialising beyond the household (Chen et al., 2023).

As elucidated in our companion paper, religious rituals vary significantly in the frequency and cost of the signals they emit, which in turn leads to different reputational advantages (CaiRangDongZhi et al., 2023). Participation in distant pilgrimages, for example, can amplify multiple dimensions of reputational quality, whereas daily religious practices typically enhance only specific ones. It appears that the reception of these reputational signals is subject to gender bias. Women with higher pilgrimage scores do not experience a significant increase in reputational standing within their peer groups. This observation is consistent with the present findings, offering a partial explanation for the association between participation in distant pilgrimages and modest gender-differentiated advantages in social relationships. In addition, it appears that, especially for men, pilgrimage participation seems to be a more significant indicator of high in-degree values compared with daily practices, although a direct comparison of effect sizes between these two variables was not conducted in our analysis. The broader visibility of pilgrimages may not only signify resource investment but also enhance social visibility, thus serving as a more potent signal. Community newcomers or those establishing themselves may find such visible rituals useful in evaluating potential partners, as grand rituals efficiently disseminate and update valuable information. For instance, a study in South India (Power, 2017a) indicates that younger religious practitioners derive greater reputational benefits from participating in dramatic annual rituals compared with their older counterparts, possibly owing to more significant information updates about these less-known individuals, making their recent religious activities more informative. The overt nature of pilgrimages, in contrast to the subtler signalling of daily practices, suggests that factors beyond cost disparity might explain why daily practices seem to play a relatively limited role in achieving high in-degree values. For future studies, comparing pilgrimages with other religious activities of similar visibility in terms of costliness, or contrasting activities of different visibility but similar costs, may provide deeper insights into the effectiveness of the scale and strength of religious signalling.

Why do women seem to receive fewer relational rewards than men for the same level of pilgrimage participation? One reason might be that pilgrimages often signal wealth or resources. In a patrilineal, patrilocal setting where men control wealth, this signal might not benefit women as much as men. Moreover, the broader signalling of pilgrimage might help in attracting new or potential cooperative partners for men, an aspect that may be less appealing to women who often favor intimate, close relationships with acquaintances. However, in this religious society that values pilgrimages as a high commitment, women's non-participation could be seen as a lack of devotion, potentially motivating them to participate owing to the risk of reputational loss. Alternatively, women's participation in pilgrimages often occurs in a family context, where they assume roles of accompanying and caring for other family members. This might be perceived as less individually risky or less indicative of personal religious devotion, and thus their contributions may be undervalued in terms of individual religious commitment compared with men. While essential, women's contributions during long-distance pilgrimages might be seen as ancillary to the ritual itself, thereby garnering fewer direct social rewards.

Both males and females who regularly engage in daily practices tend to have a comparably higher number of social connections. However, a more nuanced examination shows that for females, this trend is particularly predominant in behavioural and emotional relationships (see Figure S9). Such intimate, long-lasting connections may be particularly advantageous for women. This observation aligns with existing research where women show a preference for dyadic or cliquish relationships (David-Barrett et al., 2015; Peperkoorn et al., 2020). The composition of female social networks is typically characterised by long-lasting social interactions among members. In such settings, signal recipients often depend on less obvious, long-lasting signals to evaluate an individual's trustworthiness and commitment to maintaining the relationship. These discreet, long-term signals, which are neither extravagant nor physically demanding, might be more effective in confirming the signal's authenticity compared with short-term, highly visible ones (Bird et al., 2018). In addition, continuous engagement in religious activities offers substantial opportunities for community interaction (Dunbar, 2021), thereby facilitating the formation and strengthening of social bonds. However, a limitation of our study is that we did not investigate the influence of religious practices on the strength of social ties for both men and women. Future direction may explore whether daily religious practices, compared with less frequent activities like pilgrimages, contribute to a greater proportion of strong ties.

We cannot disregard the possibility that wealthier individuals might find it easier to undertake distant pilgrimages and exhibit greater generosity, thereby probably receiving more requests for assistance from their peers, although the economic rank was under consideration in our analysis. The informant-based wealth ranking, while apt for our study where self-reporting wealth is sensitive and standard economic measures like income or assets are challenging to quantify, may lack objectivity. Furthermore, while the three-tiered ranking system is practical for our study's purposes, it could potentially oversimplify nuances in wealth distribution and fail to capture intra-tier variations. Wealth might partly explain the gendered preferences in religious participation as well. In a society where men predominantly control wealth, women may have fewer resources for independent pilgrimages, leading them to engage more in less costly religious practices.

Men predominantly occupy central roles in our social networks, a pattern that appears to be strongly influenced by their dominant status in this patrilineal village. In such settings, the dominant gender frequently benefits from increased authority and social support (Vullioud et al., 2019). In contrast, recent research has revealed a divergent pattern in matrilineal societies, where the prominence of men is less emphasised and women are often found to have greater social support (Mattison et al., 2021). This underscores the impact of control over wealth and familial resources on the structuring of gendered social networks. Contrary to the pattern observed in other societies where households were more likely to help their neighbours (Koster & Leckie, 2014; Kasper & Mulder, 2015; Schnegg, 2015), we did not find that cooperation is constrained by spatial distance in this village, although it should be noted that we were only looking at one village so distances between household are not large. One reason for this may be the market integration that our study area has been undergoing. For instance, individuals may request advice or financial assistance via cellphones. Modern technology, such as the mobile phone and social media, has facilitated an increase in the capacity to sustain connections owing to the modernisation of the economy.

Overall, our finds shed new light on the nuanced interplay between religious rituals and social network dynamics, particularly the inherent gender disparities. This may help us develop a more comprehensive perspective on the mechanisms driving gender inequality within communities and societies.

## Conclusions

Religious involvement appears to contribute to the development of social support relationships, although the extent of benefits from personal networks might vary by gender. For infrequent, resource-intensive religious activities such as pilgrimages, a higher level of investment is associated with greater social network benefits for males more than for females, despite similar levels of commitment to these rituals by both genders. In the case of daily, low-cost religious practices that require ongoing participation, it seems that both males and females experience increased benefits at a similar rate with regular engagement.

## Supporting information

Ge et al. supplementary material 1 Ge et al. supplementary material

Ge et al. supplementary material 2 Ge et al. supplementary material

Ge et al. supplementary material 3 Ge et al. supplementary material

## Data Availability

n/a

## Supplementary material

To view supplementary material for this article, please visit https://doi.org/10.1017/ehs.2024.16

## Research transparency and reproducibility

All the R scripts and data required to replicate our analyses are available on GitHub: https://github.com/erhaoGe/Religiosity-and-gender-bias-structure-social-networks-in-a-Tibetan-population.git.
