# Supplementary material for: Religiosity and gender bias structure social networks
Source: Evol Hum Sci. 2024 Apr 8;6:e23. doi: 10.1017/ehs.2024.16 (PMC7615913; doi:10.1017/ehs.2024.16)
Supplement: Ge et al. supplementary material 1 — Ge et al. supplementary material [file S2513843X24000161sup001.docx]

**Supplementary Information for:**

**“****Religiosity and gender bias structure social networks”**

Erhao Ge ^1*^, CaiRangDongZhi ^1,2^, Ruth Mace ^1,3^

1 Department of Anthropology, University College London, 14 Taviton Street, University College London, United Kingdom

2 State Key Laboratory of Grassland and Agro-ecosystems, College of Ecology, Lanzhou University, 222 Tianshui South Rd, Lanzhou, Gansu Province, 730000, People's Republic of China

3 IAST, Toulouse School of Economics, Toulouse, Occitanie, 31080, France

* Corresponding author, email: [erhao.ge.20@ucl.ac.uk](mailto:erhao.ge.20@ucl.ac.uk)

# Supplementary Information

**Pilgrimage score**

We interviewed residents to document their history of distant pilgrimages from birth to the present, obtaining four key metrics: total number of pilgrimages in their lifetime, from their 18 to the present, over the past 10 years, and over the past 5 years. We noted that children often accompany their parents on pilgrimages, but independent pilgrimage decisions typically occur after reaching financial independence. Therefore, we excluded pilgrimage participation before the age of 18. To address recall accuracy, which is clearer for recent events, we focused on the 5-year metric for assessing personal pilgrimage costs rather than 10-year time-span participation. We then employed the following formula to measure each individual’s investment in distant pilgrimages:

$Pilgrimage score=\sum_{i=1}^{7} W_{i}\times f_{i}$ (1)

An individual's pilgrimage score is determined by summing the scores of their activities across each pilgrimage category ($i\in[1,7]$, as outlined in Table S2). The score for each category is calculated by multiplying the frequency of activities performed by the individual in that category, $f_{i}$, by the corresponding weight $W_{i}$ assigned to the category. For further details, refer to the Methods section.

**Daily practice**

To assess the regularity of practice of other low-cost, routine religious activities, we asked residents the following self-report questions:

1. *Have you visited local monasteries regularly in the past year? If yes, we then asked: How frequently did you visit? Was it on a daily, weekly, or monthly basis?*
2. *Did you perform prostrations at home every day in the past year?*
3. *Did you count prayer beads or chant sutras every day in the past year?*

*(Note: As sutra chanting is usually performed while counting beads, we combined these two activities into one question.)*

We asked interviewees for a binary response about their regular participation in the mentioned practices, rather than asking for a continuous count of frequency. This measure was chosen because, firstly, these practices are somewhat personal customs, and our primary interest was in whether individuals engage in any of these routines. Secondly, quantifying the level of participation among those who regularly perform these rituals is difficult. Many residents, especially the elderly, either do not follow a set routine, practicing whenever they can during the day, or struggle to recall the exact frequency of their practices. While some engage in these practices regularly, they might not follow a fixed schedule, and others recall only the duration of their practice, not the specific frequency. Additionally, comparing frequencies between different daily practices poses a challenge. For instance, the significance of a single home prostration differs from that of one visit to a local monastery.

It should be noted that regarding the first question, we inquired about the frequency of engagement from those who reported regular participation and discovered that all residents who engage regularly in this practice do so daily. Thus, we have categorized the data for this activity based on whether it is performed daily. For the second and third questions, some residents might not engage in these practices every day due to illness, business in town, events, or other commitments. However, if they generally adhere to a daily routine, we consider this as daily practice.

# Supplementary Tables

Table S1. Demographic characteristics of the 289 residents.

| Variables | Description | N | Mean (SD) | No. of levels |
| --- | --- | --- | --- | --- |
| Gender | Male, Female | 142 Male and 147 Female individuals | - | 2 |
| Age cohort | 16-25; 26-35; 36-45;  46-55; >55 years old. | 31; 42; 87; 63; 66 individuals respectively | - | 5 |
| Number of relatives | Number of consanguineal kin living in the village | - | 15.6 (10.6) | - |
| Communities | Named A, B, C, D, respectively | 27 A, 38 B, 21 C, 35 D households | - | 4 |
| Economic rank | Low; Medium; High. | 50 Low, 43 Medium, 28 High level households | - | 3 |
| Daily practice | Yes; No. (Ref: No) | 111 No; 177 Yes | - | - |
| Pilgrimage score | Measure for pilgrimage acts | - | 11.98 (18.53) | - |
|  |  |  |  |  |

Table S2. The consensus analysis yields ratings in terms of "Physical Cost", "Monetary Expenditure", and "Time Consumption" for each category of pilgrimage activities, classified based on the geographical distribution of monasteries, as well as for daily practices. The highest rating was designated with a value of 5, while the lowest rating was assigned a value of 1.

|  | **Physical**  **Cost** | **Monetary Expenditure** | **Time Consumption** | **Weighting score** |
| --- | --- | --- | --- | --- |
| **Pilgrimage** |  |  |  |  |
| Monastery 1 | 5 | 5 | 5 | 15 |
| Monastery 2 | 1 | 2 | 1 | 4 |
| Monastery 3 | 1 | 2 | 1 | 4 |
| Monastery 4 | 1 | 2 | 1 | 4 |
| Monastery 5 | 3 | 3 | 3 | 9 |
| Holy Mountain 1 | 5 | 5 | 5 | 15 |
| Holy Mountain 2 | 5 | 5 | 5 | 15 |
|  |  |  |  |  |
| **Daily practice** |  |  |  |  |
| Daily visiting a local monastery | 1 | 1 | 2 |  |
| Daily home prostrations | 1 | 1 | 1 |  |
| Daily bead counting and sutra recitation | 1 | 1 | 1 |  |

Table S3. Specific Types of Daily Practices by Gender.

|  | Female | Male |
| --- | --- | --- |
|  | 142 | 147 |
| Daily visiting a local monastery | 45 (31.7 %) | 10 (6.8 %) |
| Daily home prostrations | 84 (59.2 %) | 19 (12.9 %) |
| Daily bead counting and sutra recitation | 95 (66.9 %) | 64 (43.5 %) |

Table S3. The survey questions (in English and Tibetan) were used to elicit support relationships for males and females.

a)

Male’s Social Network

སྐྱེས་པའི་སྤྱི་འབྲེལ་དྲ་རྒྱ།

**Emotional Support and Friendship**

**བརྩེ་བས་རྒྱབ་སྐྱོར་དང་མཛའ་གཅུགས།**

In your village, who do you often chat with? (Whom do you want to have a casual chat with when you feel upset?)

ཁྱེད་ཚོ་སྡེ་བའི་ནང་དུ་ཁྱེད་ཀྱིས་རྒྱུན་དུ་སུ་བཙལ་ནས་ཁ་བརྡ་བྱེད་བཞིན་ཡོད་དམ།（ཁ་བརྡ་གློད་པའམ་སེམས་ཁམས་མི་སྐྱིད་དུས་མི་ཞིག་བཙལ་ནས་སྐད་ཆ་བཤད་འདོད་པ་ཡིན་ནམ།）

In your village, who are your very close friends?

ཁྱེད་ཚོ་སྡེ་བའི་ནང་དུ་ཁྱེད་ཀྱི་གྲོགས་པོ་བཟང་བོ་སུ་ཡིན་ནམ།

**Behavioural Assistance**

ངལ་རྩོལ་ནུས་ཤུགས་ཀྱི་རོགས་རམ།

In your village, who helps you to do the farm work?

ཁྱེད་ཚོ་སྡེ་བའི་ནང་དུ་མི་སུ་ཞིག་གིས་ཁྱེད་ལ་ཞིང་ལས་ལས་རོགས་བྱེད་དམ།

In your village, from whom do you seek help when your family holds a wedding or funeral?

ཁྱེད་ཚོ་སྡེ་བའི་ནང་དུ་མི་སུ་ཞིག་གིས་ཁྱེད་ཀྱི་ཁྱིམ་དུ་མནའ་མ་ལེན་པ་དང་འདས་པོར་གཤིན་ཆོས་སྒྲུབ་དུས་རོགས་རམ་བྱེད་དམ།

**Financial Assistance**

དཔལ་འབྱོར་གྱི་རོགས་འདེགས།

In your village, who do you often borrow money from when you need an amount of money?

ཁྱེད་ཚོ་སྡེ་བའི་ནང་དུ་མི་སུ་ཞིག་གིས་ཁྱེད་ལ་སྒོར་མོ་བསྐྱི་བཞིན་ཡོད་དམ།

**Guidance Assistance**

བསམ་འཆར་དང་ཁྲིད་སྟོན།

In your village, who would you like to talk with if you need to seek advice for some important things (For example: choosing a school for your children)?

ཁྱེད་ཚོ་སྡེ་བའི་ནང་དུ་ཁྱེད་ཀྱིས་སུ་ཞིག་བཙལ་ནས་རང་གིས་བལྟས་ན་དོན་དག་གལ་ཆེན་རེད་འདོད་པ་དག་ལ（དཔེར་ན། བྱིས་པ་སློབ་གྲྭ་གང་དུ་འགྲོ་བ་ལྟ་བུ།）བསམ་འཆར་རམ་བློ་བཀོད་འདོན་རོགས་བྱེད་དུ་བཅུག་པ་ཡིན་ནམ།

**Guarantee Assistance**

ཁག་ཐེག་རང་བཞིན་གྱི་ལས་ཀ

In your village, who had sought a wage labor for you?

ཁྱེད་ཚོ་སྡེ་བའི་ནང་དུ་ཁྱེད་སྒོ་ཕྱི་ལ་སོང་སྟེ་ཞོར་ལས་ལས་དུས། མི་སུ་ཞིག་གིས་ཁྱེད་ལ་ལས་ཀ་བཙལ་རོགས་བྱས་པ་ཡིན་ནམ།

b)

Female’s Social Network

བུད་མེད་ཀྱི་སྤྱི་འབྲེལ་དྲ་རྒྱ།

**Emotional Support and Friendship**

བརྩེ་བས་རྒྱབ་སྐྱོར་དང་མཛའ་གཅུགས།

In your village, who do you often chat with? (Whom do you want to have a casual chat with when you feel upset?)

ཁྱེད་ཚོ་སྡེ་བའི་ནང་དུ་ཁྱེད་ཀྱིས་རྒྱུན་དུ་སུ་བཙལ་ནས་ཁ་བརྡ་བྱེད་བཞིན་ཡོད་དམ།（ཁ་བརྡ་གློད་པའམ་སེམས་ཁམས་མི་སྐྱིད་དུས་མི་ཞིག་བཙལ་ནས་སྐད་ཆ་བཤད་འདོད་པ་ཡིན་ནམ།）

In your village, who are your very close friends?

ཁྱེད་ཚོ་སྡེ་བའི་ནང་དུ་ཁྱེད་ཀྱི་གྲོགས་པོ་བཟང་བོ་སུ་ཡིན་ནམ།

**Behavioural Assistance**

ངལ་རྩོལ་ནུས་ཤུགས་ཀྱི་རོགས་རམ།

In your village, who often help you to take care of your children?

ཁྱེད་ཚོ་སྡེ་བའི་ནང་དུ་སུ་ཞིག་གིས་དུས་རྒྱུན་དུ་ཁྱེད་ཀྱི་བྱིས་པ་ལ་བལྟ་རོགས་བྱའམ།

In your village, who do you borrow household items from?

ཁྱེད་ཚོ་སྡེ་བའི་ནང་དུ་ཁྱེད་ཀྱིས་ནམ་རྒྱུན་སུ་ཞིག་བཙལ་ཏེ་ཁྱིམ་ཆས་གཡོར་བ་ཡིན་ནམ།

In your village, who helps you to do some household chores?

ཁྱེད་ཚོ་སྡེ་བའི་ནང་དུ་ཁྱེད་ཀྱིས་ནམ་རྒྱུན་སུ་ཞིག་བཙལ་ནས་ཁྱེད་དང་མཉམ་དུ་ལས་ཀ་ཙག་ཙིག་བྱེད་ཀྱིན་ཡོད་དམ། དཔེར་ན། གོ་རེ་བཙོ་བ་ལྟ་བུ།

In your village, from whom do you seek help when your family holds a wedding or funeral?

ཁྱེད་ཚོ་སྡེ་བའི་ནང་དུ་མི་སུ་ཞིག་གིས་ཁྱེད་ཀྱི་ཁྱིམ་དུ་མནའ་མ་ལེན་པ་དང་འདས་པོར་གཤིན་ཆོས་སྒྲུབ་དུས་རོགས་རམ་བྱེད་དམ།

**Financial Assistance**

དཔལ་འབྱོར་གྱི་རོགས་འདེགས།

In your village, who do you often borrow money from when you need an amount of money?

ཁྱེད་ཚོ་སྡེ་བའི་ནང་དུ་མི་སུ་ཞིག་གིས་ཁྱེད་ལ་སྒོར་མོ་བསྐྱི་བཞིན་ཡོད་དམ།

**Guidance Assistance**

བསམ་འཆར་དང་ཁྲིད་སྟོན།

In your village, who do you seek advice for some important things that you cannot handle?

ཁྱེད་ཚོ་སྡེ་བའི་ནང་དུ་ཁྱེད་ཀྱིས་རྒྱུན་དུ་སུ་ཞིག་བཙལ་ནས་རང་གིས་དོན་གལ་ཆེན་རེད་འདོད་པ་དག་ལ་ཁ་བརྡ་བྱེད་བཞིན་ཡོད་དམ། ཡང་ན་བསམ་འཆར་རམ་བློ་བཀོད་འདོན་རོགས་བྱེད་དུ་བཅུག་པ་ཡིན་ནམ།

**Guarantee Assistance**

ཁག་ཐེག་རང་བཞིན་གྱི་ལས་ཀ

In your village, who had sought a wage labor for you?

ཁྱེད་ཚོ་སྡེ་བའི་ནང་དུ་ཁྱེད་སྒོ་ཕྱི་ལ་སོང་སྟེ་ཞོར་ལས་ལས་དུས། མི་སུ་ཞིག་གིས་ཁྱེད་ལ་ལས་ཀ་བཙལ་རོགས་བྱས་པ་ཡིན་ནམ།

Table S4. Summary of the nominations given and received by gender.

|  | Gender | Average Number of Nominations per Nominator | Average Number of Nominees per Nominator | Average Proportion of  Male Nominees | Average Proportion of  Female Nominees |
| --- | --- | --- | --- | --- | --- |
| Nominator  287 | Male  147 | 13.7(5.05) | 9.97(3.81) | 0.96 (0.10) | 0.04 (0.10) |
|  | Female  140 | 15.7(5.10) | 11.1(3.67) | 0.36 (0.19) | 0.64 (0.19) |
|  |  |  |  |  |  |

|  | Gender | Average  Number of Nominations per  Nominee | Average  Number of Nominators per  Nominee | Average Proportion of  Male Nominators | Average Proportion of  Female Nominators |
| --- | --- | --- | --- | --- | --- |
| Nominee  272 | Male  142 | 18.2(16.1) | 14.0(12.9) | 0.75(0.24) | 0.25(0.24) |
|  | Female 130 | 12.5(8.49) | 7.9(5.24) | 0.06(0.14) | 0.94(0.14) |

Table S5. Summary statistics of sociocentric networks for each type of social support.

|  | Full | Emotional | Behavioural | Guidance | Financial | Guarantee |
| --- | --- | --- | --- | --- | --- | --- |
| Edges | 4214 | 1559 | 1510 | 513 | 333 | 299 |
| Nodes | 288 | 285 | 287 | 286 | 274 | 243 |
| Mean degree | 29.162 | 10.940 | 10.522 | 3.587 | 2.430 | 2.461 |
| Mean  In/Out degree | 14.581 | 5.470 | 5.261 | 1.794 | 1.215 | 1.230 |
| Density | 0.051 | 0.019 | 0.018 | 0.006 | 0.004 | 0.005 |
| Reciprocity | 0.303 | 0.348 | 0.262 | 0.105 | 0.168 | 0.080 |
| Transitivity | 0.199 | 0.239 | 0.181 | 0.210 | 0.103 | 0.075 |
| Diameter | 10 | 21 | 16 | 10 | 12 | 10 |

Table S6. Description of the variables used in the exponential random graph models. Node terms capture the influence of individual attributes (nodes) on the likelihood of forming support ties (edges). Edge terms examine the effects of various relationships between pairs of individuals (dyads) on tie formation, e.g., gender homophily and geographical proximity. “In” terms refer to variables influencing incoming ties, which represent individuals who are nominated as providers of support. Covariates refer to numeric predictors, while factors denote categorical variables.

| Variable | Term Type | Description |
| --- | --- | --- |
| Age | Node in-covariate | Individual’s age |
| Gender | Node in-factor | Individual’s gender |
| Economic Rank | Node in-factor | The economic rank of the household where the individual resides |
| Same Community | Edge factor | Whether two individuals are of the same community |
| Same Gender | Edge factor | Whether two individuals have the same gender |
| Relatedness | Edge covariate | Consanguineous relatedness between two individuals |
| Affinal Relatedness | Edge covariate | Affinal relatedness between two individuals |
| Geographic Distance | Edge covariate | Distance (measured in meters) between individuals' houses. |
| Pilgrimage Score | Node in-covariate | A tally of the pilgrimage acts performed over a 5-year period. |
| Daily Practice | Node in-factor | Whether or not an individual participates in daily religious practices regularly. |
| Reciprocity | Network statistic | The number of pairs in which a reciprocal tie exists |
| GWDSP | Network statistic | Geometrically weighted dyad-wise shared partners. The number of partners held in common by two individuals. |
| In-Degree (0) | Network statistic | The number of nodes without incoming ties, i.e., individuals who have never been nominated as providers of the particular type of support. |
| Out-Degree (0) | Network statistic | The number of nodes without outgoing ties, i.e., individuals who have never nominated others for support. |

Table S6. Stepwise exponential random graph models predicting the log odds of a tie in the full supportive network. Model 3 is the main model

reported in Table 1.

|  | Control | | | Control + religiosity | | | Control + religiosity + Sturcture | | |
| --- | --- | --- | --- | --- | --- | --- | --- | --- | --- |
| **Variables** | Estimate | 95% CI | *p*-value | Estimate | 95% CI | *p*-value | Estimate | 95% CI | *p*-value |
| **Edges** | -5.29 | -5.53, -5.06 | <0.001 | -5.50 | -5.74, -5.26 | <0.001 | -4.53 | -4.83, -4.24 | <0.001 |
| Age (unit: Year) | 0.013 | 0.010, 0.016 | <0.001 | 0.012 | 0.009, 0.016 | <0.001 | 0.012 | 0.008, 0.016 | <0.001 |
| Gender (Male; Ref: Female) | 0.723 | 0.618, 0.828 | <0.001 | 0.750 | 0.636, 0.865 | <0.001 | 1.00 | 0.871, 1.13 | <0.001 |
| Economic Rank (Low; Ref: High) | -0.124 | -0.243, -0.006 | 0.040 | 0.001 | -0.123, 0.125 | >0.9 | 0.012 | -0.110, 0.135 | 0.8 |
| Economic Rank (Middle; Ref: High) | -0.279 | -0.401, -0.157 | <0.001 | -0.143 | -0.270, -0.015 | 0.028 | -0.135 | -0.260, -0.010 | 0.034 |
| Same Community | 1.22 | 1.13, 1.32 | <0.001 | 1.23 | 1.13, 1.33 | <0.001 | 0.920 | 0.836, 1.00 | <0.001 |
| Same Gender | 1.19 | 1.08, 1.30 | <0.001 | 1.19 | 1.08, 1.31 | <0.001 | 1.00 | 0.900, 1.11 | <0.001 |
| Relatedness | 1.54 | 0.983, 2.09 | <0.001 | 1.58 | 1.03, 2.13 | <0.001 | 1.08 | 0.602, 1.55 | <0.001 |
| Affinal Relatedness | 0.073 | 0.001, 0.145 | 0.048 | 0.071 | -0.002, 0.143 | 0.055 | 0.047 | -0.015, 0.108 | 0.13 |
| Geographic Distance (unit: Meter) | -0.004 | -0.005, -0.004 | <0.001 | -0.004 | -0.005, -0.004 | <0.001 | -0.004 | -0.004, -0.003 | <0.001 |
| Pilgrimage Score |  |  |  | 0.152 | 0.115, 0.189 | <0.001 | 0.127 | 0.093, 0.162 | <0.001 |
| Daily Practice (Yes; Ref: No) |  |  |  | 0.178 | 0.055, 0.301 | 0.005 | 0.158 | 0.037, 0.279 | 0.010 |
| **Structure Terms** |  |  |  |  |  |  |  |  |  |
| Reciprocity |  |  |  |  |  |  | 3.24 | 3.06, 3.43 | <0.001 |
| GWDSP (α = 0.5) |  |  |  |  |  |  | -0.127 | -0.142, -0.111 | <0.001 |
| **AIC** |  | 15051 |  |  | 14984 |  |  | 13898 |  |

Table S7. Stepwise exponential random graph models predicting the log odds of a tie in the emotional support network.

|  | Control | | | Control + religiosity | | | Control + religiosity + Sturcture | | |
| --- | --- | --- | --- | --- | --- | --- | --- | --- | --- |
| Variables | Estimate | 95% CI | *p*-value | Estimate | 95% CI | *p*-value | Estimate | 95% CI | *p*-value |
| Edges | -7.74 | -8.29, -7.19 | <0.001 | -7.82 | -8.37, -7.27 | <0.001 | -7.24 | -7.81, -6.67 | <0.001 |
| Age (unit: Years) | -0.001 | -0.006, 0.004 | 0.7 | -0.002 | -0.007, 0.004 | 0.5 | -0.001 | -0.006, 0.004 | 0.6 |
| Gender (Male; Ref: Female) | 0.092 | -0.050, 0.235 | 0.2 | 0.110 | -0.048, 0.268 | 0.2 | 0.136 | -0.028, 0.301 | 0.10 |
| Economic Rank  (Low; Ref: High) | 0.046 | -0.131, 0.222 | 0.6 | 0.103 | -0.080, 0.286 | 0.3 | 0.084 | -0.083, 0.250 | 0.3 |
| Economic Rank (Middle; Ref: High) | -0.102 | -0.288, 0.084 | 0.3 | -0.038 | -0.231, 0.155 | 0.7 | -0.029 | -0.200, 0.143 | 0.7 |
| Same Community | 0.448 | 0.301, 0.596 | <0.001 | 0.449 | 0.301, 0.597 | <0.001 | 0.332 | 0.205, 0.459 | <0.001 |
| Same Gender | 3.72 | 3.26, 4.17 | <0.001 | 3.72 | 3.26, 4.17 | <0.001 | 3.31 | 2.85, 3.76 | <0.001 |
| Relatedness | 0.667 | -0.186, 1.52 | 0.13 | 0.666 | -0.186, 1.52 | 0.13 | 0.491 | -0.251, 1.23 | 0.2 |
| Affinal Relatedness | 0.087 | -0.015, 0.189 | 0.095 | 0.087 | -0.016, 0.189 | 0.10 | 0.062 | -0.028, 0.152 | 0.2 |
| Geographic Distance (unit: Meter) | 0.000 | -0.001, 0.000 | 0.5 | 0.000 | -0.001, 0.000 | 0.5 | 0.000 | -0.001, 0.000 | 0.5 |
| Pilgrimage Score |  |  |  | 0.081 | 0.017, 0.144 | 0.013 | 0.068 | 0.007, 0.129 | 0.028 |
| Daily Practice  (Yes; Ref: No) |  |  |  | 0.091 | -0.087, 0.268 | 0.3 | 0.070 | -0.089, 0.230 | 0.4 |
| Reciprocity |  |  |  |  |  |  | 3.80 | 3.55, 4.05 | <0.001 |
| In-Degree (0) |  |  |  |  |  |  | 1.70 | 1.27, 2.13 | <0.001 |
| Out-Degree (0) |  |  |  |  |  |  | -3.00 | -4.98, -1.02 | 0.003 |
| GWDSP (α = 0.5) |  |  |  |  |  |  | -0.098 | -0.145, -0.052 | <0.001 |
| **AIC** | 7955.683 |  |  |  | 7952.429 |  |  | 7237.580 |  |

Table S8. Stepwise exponential random graph models predicting the log odds of a tie in the behavioural support network.

|  | Control | | | Control + religiosity | | | Control + religiosity + Sturcture | | |
| --- | --- | --- | --- | --- | --- | --- | --- | --- | --- |
| Variables | Estimate | 95% CI | p-value | Estimate | 95% CI | p-value | Estimate | 95% CI | p-value |
| Edges | -6.28 | -6.59, -5.97 | <0.001 | -6.53 | -6.85, -6.21 | <0.001 | -5.96 | -6.33, -5.60 | <0.001 |
| Age (unit: Years) | 0.011 | 0.007, 0.015 | <0.001 | 0.009 | 0.004, 0.013 | <0.001 | 0.007 | 0.003, 0.012 | 0.001 |
| Gender (Male; Ref: Female) | 0.299 | 0.174, 0.423 | <0.001 | 0.351 | 0.215, 0.487 | <0.001 | 0.301 | 0.169, 0.433 | <0.001 |
| Economic Rank  (Low; Ref: High) | 0.054 | -0.098, 0.205 | 0.5 | 0.195 | 0.035, 0.355 | 0.017 | 0.164 | 0.016, 0.312 | 0.030 |
| Economic Rank (Middle; Ref: High) | -0.132 | -0.290, 0.026 | 0.10 | 0.022 | -0.144, 0.188 | 0.8 | 0.020 | -0.135, 0.175 | 0.8 |
| Same Community | 1.26 | 1.14, 1.38 | <0.001 | 1.26 | 1.14, 1.38 | <0.001 | 0.993 | 0.885, 1.10 | <0.001 |
| Same Gender | 1.29 | 1.15, 1.44 | <0.001 | 1.30 | 1.15, 1.44 | <0.001 | 1.06 | 0.921, 1.20 | <0.001 |
| Relatedness | 1.22 | 0.501, 1.94 | <0.001 | 1.26 | 0.538, 1.97 | <0.001 | 0.945 | 0.335, 1.56 | 0.002 |
| Affinal Relatedness | 0.102 | 0.013, 0.191 | 0.025 | 0.100 | 0.011, 0.190 | 0.028 | 0.072 | -0.005, 0.148 | 0.067 |
| Geographic Distance (unit: Meter) | 0.000 | -0.001, 0.000 | 0.6 | 0.000 | -0.001, 0.001 | 0.8 | 0.000 | -0.001, 0.001 | 0.8 |
| Pilgrimage Score |  |  |  | 0.166 | 0.118, 0.214 | <0.001 | 0.159 | 0.110, 0.207 | <0.001 |
| Daily Practice  (Yes; Ref: No) |  |  |  | 0.262 | 0.107, 0.417 | <0.001 | 0.222 | 0.079, 0.365 | 0.002 |
| Reciprocity |  |  |  |  |  |  | 3.38 | 3.15, 3.61 | <0.001 |
| In-Degree (0) |  |  |  |  |  |  | 1.72 | 1.24, 2.20 | <0.001 |
| Out-Degree (0) |  |  |  |  |  |  | -1.26 | -2.69, 0.163 | 0.083 |
| GWDSP (α = 0.5) |  |  |  |  |  |  | -0.054 | -0.082, -0.025 | <0.001 |
| **AIC** |  | 10643.869 |  |  | 10592.775 |  |  | 9937.08 |  |

Table S9. Stepwise exponential random graph models predicting the log odds of a tie in the guidance support network.

|  | Control | | | Control + religiosity | | | Control + religiosity + Sturcture | | |
| --- | --- | --- | --- | --- | --- | --- | --- | --- | --- |
| Variables | Estimate | 95% CI | p-value | Estimate | 95% CI | p-value | Estimate | 95% CI | p-value |
| Edges | -8.35 | -8.83, -7.86 | <0.001 | -8.67 | -9.17, -8.17 | <0.001 | -7.50 | -8.08, -6.93 | <0.001 |
| Age (unit: Years) | 0.033 | 0.027, 0.038 | <0.001 | 0.031 | 0.024, 0.037 | <0.001 | 0.023 | 0.017, 0.030 | <0.001 |
| Gender (Male; Ref: Female) | 2.10 | 1.80, 2.40 | <0.001 | 2.15 | 1.84, 2.47 | <0.001 | 1.40 | 1.11, 1.70 | <0.001 |
| Economic Rank  (Low; Ref: High) | -0.760 | -0.986, -0.534 | <0.001 | -0.568 | -0.804, -0.333 | <0.001 | -0.409 | -0.614, -0.204 | <0.001 |
| Economic Rank (Middle; Ref: High) | -0.602 | -0.807, -0.396 | <0.001 | -0.412 | -0.627, -0.196 | <0.001 | -0.304 | -0.492, -0.117 | 0.001 |
| Same Community | 2.14 | 1.94, 2.34 | <0.001 | 2.14 | 1.94, 2.34 | <0.001 | 2.03 | 1.83, 2.23 | <0.001 |
| Same Gender | 0.236 | 0.053, 0.419 | 0.011 | 0.240 | 0.057, 0.424 | 0.010 | 0.426 | 0.197, 0.654 | <0.001 |
| Relatedness | 2.18 | 1.22, 3.13 | <0.001 | 2.28 | 1.32, 3.23 | <0.001 | 1.96 | 1.10, 2.82 | <0.001 |
| Affinal Relatedness | 0.174 | 0.051, 0.296 | 0.005 | 0.166 | 0.043, 0.288 | 0.008 | 0.142 | 0.033, 0.251 | 0.011 |
| Geographic Distance (unit: Meter) | -0.004 | -0.005, -0.003 | <0.001 | -0.004 | -0.005, -0.003 | <0.001 | -0.004 | -0.005, -0.003 | <0.001 |
| Pilgrimage Score |  |  |  | 0.188 | 0.133, 0.244 | <0.001 | 0.156 | 0.102, 0.210 | <0.001 |
| Daily Practice  (Yes; Ref: No) |  |  |  | 0.324 | 0.091, 0.556 | 0.006 | 0.248 | 0.051, 0.445 | 0.014 |
| Reciprocity |  |  |  |  |  |  | 2.20 | 1.70, 2.69 | <0.001 |
| In-Degree (0) |  |  |  |  |  |  | 1.87 | 1.38, 2.36 | <0.001 |
| Out-Degree (0) |  |  |  |  |  |  | -3.31 | -4.33, -2.30 | <0.001 |
| GWDSP (α = 0.5) |  |  |  |  |  |  | -0.079 | -0.128, -0.030 | 0.002 |
| **AIC** |  | 4987.370 |  |  | 4941.150 |  |  | 4723.151 |  |

Table S10. Stepwise exponential random graph models predicting the log odds of a tie in the financial support network.

|  | Control | | | Control + religiosity | | | Control + religiosity + Sturcture | | |
| --- | --- | --- | --- | --- | --- | --- | --- | --- | --- |
| Variables | Estimate | 95% CI | p-value | Estimate | 95% CI | p-value | Estimate | 95% CI | p-value |
| Edges | -7.09 | -7.64, -6.55 | <0.001 | -7.19 | -7.74, -6.63 | <0.001 | -7.76 | -8.44, -7.07 | <0.001 |
| Age (unit: Years) | 0.004 | -0.003, 0.012 | 0.3 | 0.003 | -0.006, 0.012 | 0.5 | 0.003 | -0.005, 0.011 | 0.5 |
| Gender (Male; Ref: Female) | 0.602 | 0.367, 0.836 | <0.001 | 0.617 | 0.360, 0.874 | <0.001 | 0.770 | 0.466, 1.07 | <0.001 |
| Economic Rank  (Low; Ref: High) | -1.07 | -1.35, -0.794 | <0.001 | -1.01 | -1.29, -0.722 | <0.001 | -0.808 | -1.08, -0.538 | <0.001 |
| Economic Rank (Middle; Ref: High) | -0.653 | -0.906, -0.400 | <0.001 | -0.578 | -0.840, -0.316 | <0.001 | -0.476 | -0.723, -0.230 | <0.001 |
| Same Community | 1.21 | 0.994, 1.43 | <0.001 | 1.21 | 0.994, 1.43 | <0.001 | 1.05 | 0.846, 1.26 | <0.001 |
| Same Gender | 1.38 | 1.10, 1.65 | <0.001 | 1.38 | 1.11, 1.65 | <0.001 | 1.44 | 1.13, 1.74 | <0.001 |
| Relatedness | 1.34 | 0.165, 2.51 | 0.025 | 1.36 | 0.187, 2.53 | 0.023 | 1.06 | 0.009, 2.11 | 0.048 |
| Affinal Relatedness | 0.200 | 0.063, 0.336 | 0.004 | 0.197 | 0.060, 0.334 | 0.005 | 0.160 | 0.034, 0.285 | 0.013 |
| Geographic Distance (unit: Meter) | 0.001 | 0.000, 0.002 | 0.018 | 0.001 | 0.000, 0.003 | 0.014 | 0.001 | 0.000, 0.002 | 0.015 |
| Pilgrimage Score |  |  |  | 0.093 | 0.014, 0.172 | 0.021 | 0.083 | 0.005, 0.160 | 0.037 |
| Daily Practice  (Yes; Ref: No) |  |  |  | 0.096 | -0.179, 0.371 | 0.5 | 0.087 | -0.166, 0.339 | 0.5 |
| Reciprocity |  |  |  |  |  |  | 3.38 | 2.88, 3.88 | <0.001 |
| In-Degree (0) |  |  |  |  |  |  | 0.781 | 0.374, 1.19 | <0.001 |
| Out-Degree (0) |  |  |  |  |  |  | -2.78 | -3.30, -2.25 | <0.001 |
| GWDSP (α = 0.5) |  |  |  |  |  |  | -0.105 | -0.234, 0.023 | 0.11 |
| **AIC** |  | 3916.789 |  |  | 3915.052 |  |  | 3632.194 |  |

Table S11. Stepwise exponential random graph models predicting the log odds of a tie in the guarantee support network.

|  | Control | | | Control + religiosity | | | Control + religiosity + Sturcture | | |
| --- | --- | --- | --- | --- | --- | --- | --- | --- | --- |
| Variables | Estimate | 95% CI | p-value | Estimate | 95% CI | p-value | Estimate | 95% CI | p-value |
| Edges | -6.56 | -7.14, -5.99 | <0.001 | -6.85 | -7.45, -6.26 | <0.001 | -6.62 | -7.27, -5.98 | <0.001 |
| Age (unit: Years) | 0.006 | -0.002, 0.013 | 0.14 | 0.006 | -0.003, 0.015 | 0.2 | 0.004 | -0.004, 0.012 | 0.3 |
| Gender (Male; Ref: Female) | 0.911 | 0.642, 1.18 | <0.001 | 0.910 | 0.619, 1.20 | <0.001 | 0.808 | 0.513, 1.10 | <0.001 |
| Economic Rank  (Low; Ref: High) | -0.826 | -1.10, -0.549 | <0.001 | -0.648 | -0.939, -0.357 | <0.001 | -0.465 | -0.728, -0.203 | <0.001 |
| Economic Rank (Middle; Ref: High) | -0.670 | -0.951, -0.388 | <0.001 | -0.476 | -0.774, -0.179 | 0.002 | -0.337 | -0.594, -0.080 | 0.010 |
| Same Community | 1.30 | 1.07, 1.53 | <0.001 | 1.30 | 1.07, 1.53 | <0.001 | 1.22 | 0.994, 1.46 | <0.001 |
| Same Gender | 0.756 | 0.506, 1.00 | <0.001 | 0.757 | 0.507, 1.01 | <0.001 | 0.882 | 0.607, 1.16 | <0.001 |
| Relatedness | 0.687 | -0.761, 2.13 | 0.4 | 0.788 | -0.655, 2.23 | 0.3 | 0.721 | -0.641, 2.08 | 0.3 |
| Affinal Relatedness | 0.122 | -0.045, 0.288 | 0.2 | 0.114 | -0.052, 0.281 | 0.2 | 0.104 | -0.051, 0.258 | 0.2 |
| Geographic Distance (unit: Meter) | 0.000 | -0.002, 0.001 | 0.5 | 0.000 | -0.001, 0.001 | 0.7 | 0.000 | -0.002, 0.001 | 0.6 |
| Pilgrimage Score |  |  |  | 0.169 | 0.098, 0.239 | <0.001 | 0.143 | 0.076, 0.210 | <0.001 |
| Daily Practice  (Yes; Ref: No) |  |  |  | 0.157 | -0.130, 0.443 | 0.3 | 0.123 | -0.123, 0.369 | 0.3 |
| Reciprocity |  |  |  |  |  |  | 2.40 | 1.74, 3.06 | <0.001 |
| In-Degree (0) |  |  |  |  |  |  | 1.02 | 0.579, 1.46 | <0.001 |
| Out-Degree (0) |  |  |  |  |  |  | -1.72 | -2.17, -1.26 | <0.001 |
| GWDSP (α = 0.5) |  |  |  |  |  |  | -0.205 | -0.318, -0.091 | <0.001 |
| **AIC** |  | 3495.248 |  |  | 3478.091 |  |  | 3356.318 |  |

Table S12. Full exponential random graph models predicting the log odds of a tie in each of the five social support type networks.

|  | Emotional | | | Behavioural | | | Guidance | | | Financial | | | Guarantee | | |
| --- | --- | --- | --- | --- | --- | --- | --- | --- | --- | --- | --- | --- | --- | --- | --- |
|  |  |  |  |  |  |  |  |  |  |  |  |  |  |  |  |
| Variables | *Est* | *95% CI* | *p* | *Est* | *95% CI* | *p* | *Est* | *95% CI* | *p* | *Est* | *95% CI* | *p* | *Est* | *95% CI* | *p* |
| Edges | -7.24 | -7.81, -6.67 | <0.001 | -5.96 | -6.33, -5.60 | <0.001 | -7.50 | -8.08, -6.93 | <0.001 | -7.76 | -8.44, -7.07 | <0.001 | -6.62 | -7.27, -5.98 | <0.001 |
| Age (unit: Years) | -0.001 | -0.006, 0.004 | 0.6 | 0.007 | 0.003, 0.012 | 0.001 | 0.023 | 0.017, 0.030 | <0.001 | 0.003 | -0.005, 0.011 | 0.5 | 0.004 | -0.004, 0.012 | 0.3 |
| Gender (Male; Ref: Female) | 0.136 | -0.028, 0.301 | 0.10 | 0.301 | 0.169, 0.433 | <0.001 | 1.40 | 1.11, 1.70 | <0.001 | 0.770 | 0.466, 1.07 | <0.001 | 0.808 | 0.513, 1.10 | <0.001 |
| Economic Rank  (Low; Ref: High) | 0.084 | -0.083, 0.250 | 0.3 | 0.164 | 0.016, 0.312 | 0.030 | -0.409 | -0.614, -0.204 | <0.001 | -0.808 | -1.08, -0.538 | <0.001 | -0.465 | -0.728, -0.203 | <0.001 |
| Economic Rank (Middle; Ref: High) | -0.029 | -0.200, 0.143 | 0.7 | 0.020 | -0.135, 0.175 | 0.8 | -0.304 | -0.492, -0.117 | 0.001 | -0.476 | -0.723, -0.230 | <0.001 | -0.337 | -0.594, -0.080 | 0.010 |
| Same Community | 0.332 | 0.205, 0.459 | <0.001 | 0.993 | 0.885, 1.10 | <0.001 | 2.03 | 1.83, 2.23 | <0.001 | 1.05 | 0.846, 1.26 | <0.001 | 1.22 | 0.994, 1.46 | <0.001 |
| Same Gender | 3.31 | 2.85, 3.76 | <0.001 | 1.06 | 0.921, 1.20 | <0.001 | 0.426 | 0.197, 0.654 | <0.001 | 1.44 | 1.13, 1.74 | <0.001 | 0.882 | 0.607, 1.16 | <0.001 |
| Relatedness | 0.491 | -0.251, 1.23 | 0.2 | 0.945 | 0.335, 1.56 | 0.002 | 1.96 | 1.10, 2.82 | <0.001 | 1.06 | 0.009, 2.11 | 0.048 | 0.721 | -0.641, 2.08 | 0.3 |
| Affinal Relatedness | 0.062 | -0.028, 0.152 | 0.2 | 0.072 | -0.005, 0.148 | 0.067 | 0.142 | 0.033, 0.251 | 0.011 | 0.160 | 0.034, 0.285 | 0.013 | 0.104 | -0.051, 0.258 | 0.2 |
| Geographic Distance (unit: Meter) | 0.000 | -0.001, 0.000 | 0.5 | 0.000 | -0.001, 0.001 | 0.8 | -0.004 | -0.005, -0.003 | <0.001 | 0.001 | 0.000, 0.002 | 0.015 | 0.000 | -0.002, 0.001 | 0.6 |
| Pilgrimage Score | 0.068 | 0.007, 0.129 | 0.028 | 0.159 | 0.110, 0.207 | <0.001 | 0.156 | 0.102, 0.210 | <0.001 | 0.083 | 0.005, 0.160 | 0.037 | 0.143 | 0.076, 0.210 | <0.001 |
| Daily Practice  (Yes; Ref: No) | 0.070 | -0.089, 0.230 | 0.4 | 0.222 | 0.079, 0.365 | 0.002 | 0.248 | 0.051, 0.445 | 0.014 | 0.087 | -0.166, 0.339 | 0.5 | 0.123 | -0.123, 0.369 | 0.3 |
| Structure Terms |  |  |  |  |  |  |  |  |  |  |  |  |  |  |  |
| Reciprocity | 3.80 | 3.55, 4.05 | <0.001 | 3.38 | 3.15, 3.61 | <0.001 | 2.20 | 1.70, 2.69 | <0.001 | 3.38 | 2.88, 3.88 | <0.001 | 2.40 | 1.74, 3.06 | <0.001 |
| In-Degree (0) | 1.70 | 1.27, 2.13 | <0.001 | 1.72 | 1.24, 2.20 | <0.001 | 1.87 | 1.38, 2.36 | <0.001 | 0.781 | 0.374, 1.19 | <0.001 | 1.02 | 0.579, 1.46 | <0.001 |
| Out-Degree (0) | -3.00 | -4.98, -1.02 | 0.003 | -1.26 | -2.69, 0.163 | 0.083 | -3.31 | -4.33, -2.30 | <0.001 | -2.78 | -3.30, -2.25 | <0.001 | -1.72 | -2.17, -1.26 | <0.001 |
| GWDSP (α = 0.5) | -0.098 | -0.145, -0.052 | <0.001 | -0.054 | -0.082, -0.025 | <0.001 | -0.079 | -0.128, -0.030 | 0.002 | -0.105 | -0.234, 0.023 | 0.11 | -0.205 | -0.318, -0.091 | <0.001 |

Table S13. Estimates from negative binomial regression models assessing various predictors of in-degree value in the personal networks of 284 individuals (Four individuals were excluded from model fitting due to missing information regarding the number of siblings in the village).

| Model determinants | | | | |
| --- | --- | --- | --- | --- |
|  | | | | |
|  | *Dependent variable:* | | | |
|  | In-degree values | | | |
|  | Control | Control+Pilgrimage+Daily practice | Control+Pilgrimage+  Gender*Daily practice | Control+Daily practice+  Gender*Pilgrimage |
|  | | | | |
| Age Cohort (16-25; Ref: >55) | -0.574** | -0.499** | -0.481** | -0.457* |
|  | (-1.025, -0.123) | (-0.963, -0.035) | (-0.949, -0.014) | (-0.921, 0.008) |
| Age Cohort (26-35; Ref: >55) | -0.370** | -0.256 | -0.262 | -0.264 |
|  | (-0.717, -0.022) | (-0.617, 0.105) | (-0.623, 0.098) | (-0.625, 0.097) |
| Age Cohort (36-45; Ref: >55) | -0.035 | 0.018 | 0.007 | 0.009 |
|  | (-0.309, 0.240) | (-0.261, 0.298) | (-0.273, 0.287) | (-0.270, 0.288) |
| Age Cohort (46-55; Ref: >55) | 0.148 | 0.186 | 0.182 | 0.186 |
|  | (-0.146, 0.442) | (-0.109, 0.482) | (-0.114, 0.477) | (-0.109, 0.481) |
| Gender (Male; Ref: Female) | 0.359**** | 0.430**** | 0.548*** | 0.433**** |
|  | (0.158, 0.559) | (0.213, 0.648) | (0.180, 0.916) | (0.216, 0.650) |
| Economic Rank (Low; Ref: High) | -0.138 | -0.051 | -0.048 | -0.071 |
|  | (-0.389, 0.113) | (-0.306, 0.203) | (-0.303, 0.206) | (-0.326, 0.184) |
| Economic Rank (Middle; Ref: High) | -0.243* | -0.131 | -0.125 | -0.136 |
|  | (-0.496, 0.009) | (-0.388, 0.126) | (-0.382, 0.131) | (-0.392, 0.120) |
| Number of siblings in the village | 0.006 | -0.004 | -0.002 | -0.002 |
|  | (-0.072, 0.084) | (-0.081, 0.073) | (-0.079, 0.075) | (-0.078, 0.075) |
| Number of offspring in the village | 0.002 | -0.033 | -0.028 | -0.032 |
|  | (-0.122, 0.126) | (-0.156, 0.091) | (-0.153, 0.096) | (-0.156, 0.091) |
| Pilgrimage score |  | 0.117** | 0.119** | 0.004 |
|  |  | (0.019, 0.215) | (0.022, 0.217) | (-0.181, 0.189) |
| Daily Practice (Yes; Ref: No) |  | 0.240* | 0.352* | 0.240* |
|  |  | (-0.005, 0.484) | (-0.024, 0.727) | (-0.003, 0.484) |
| Gender (Male):Daily Practice (Yes) |  |  | -0.179 |  |
|  |  |  | (-0.626, 0.268) |  |
| Gender (Male):Pilgrimage score |  |  |  | 0.156 |
|  |  |  |  | (-0.058, 0.369) |
| Constant | 2.671**** | 2.421**** | 2.322**** | 2.417**** |
|  | (2.308, 3.034) | (1.990, 2.852) | (1.820, 2.824) | (1.988, 2.846) |
|  | | | | |
| Observations | 284 | 284 | 284 | 284 |
|  | | | | |
| *Note:* | **p<0.1 **p<0.05 ***p<0.01 ****p<0.001* | | | |

Table S14. Model selection results from negative binomial models on in-degree value in personal networks. Columns represent the number of parameters (K), Corrected Akaike Information Criterion (AICc), AICc differences (ΔAICc), log-likelihood (LL), and cumulative weights (CUM.WT). Model names signify specific variable combinations. The Control model includes variables such as age cohort, gender, economic rank of the household, number of siblings, and number of offspring in the village.

| *Models* | *K* | *AICc* | *ΔAICc* | *LL* | *Cum.Wt* |
| --- | --- | --- | --- | --- | --- |
| Control+Pilgrimage+  Daily Practice | 13 | 2078.626 | 0 | -1025.64 | 0.422688 |
| Control+Daily Practice+  Gender*Pilgrimage | 14 | 2078.946 | 0.320345 | -1024.69 | 0.782817 |
| Control+Pilgrimage+  Gender*Daily Practice | 14 | 2080.229 | 1.60282 | -1025.33 | 0.972475 |
| Control | 11 | 2084.089 | 5.463086 | -1030.56 | 1 |

# Supplementary figures

**a)**

**
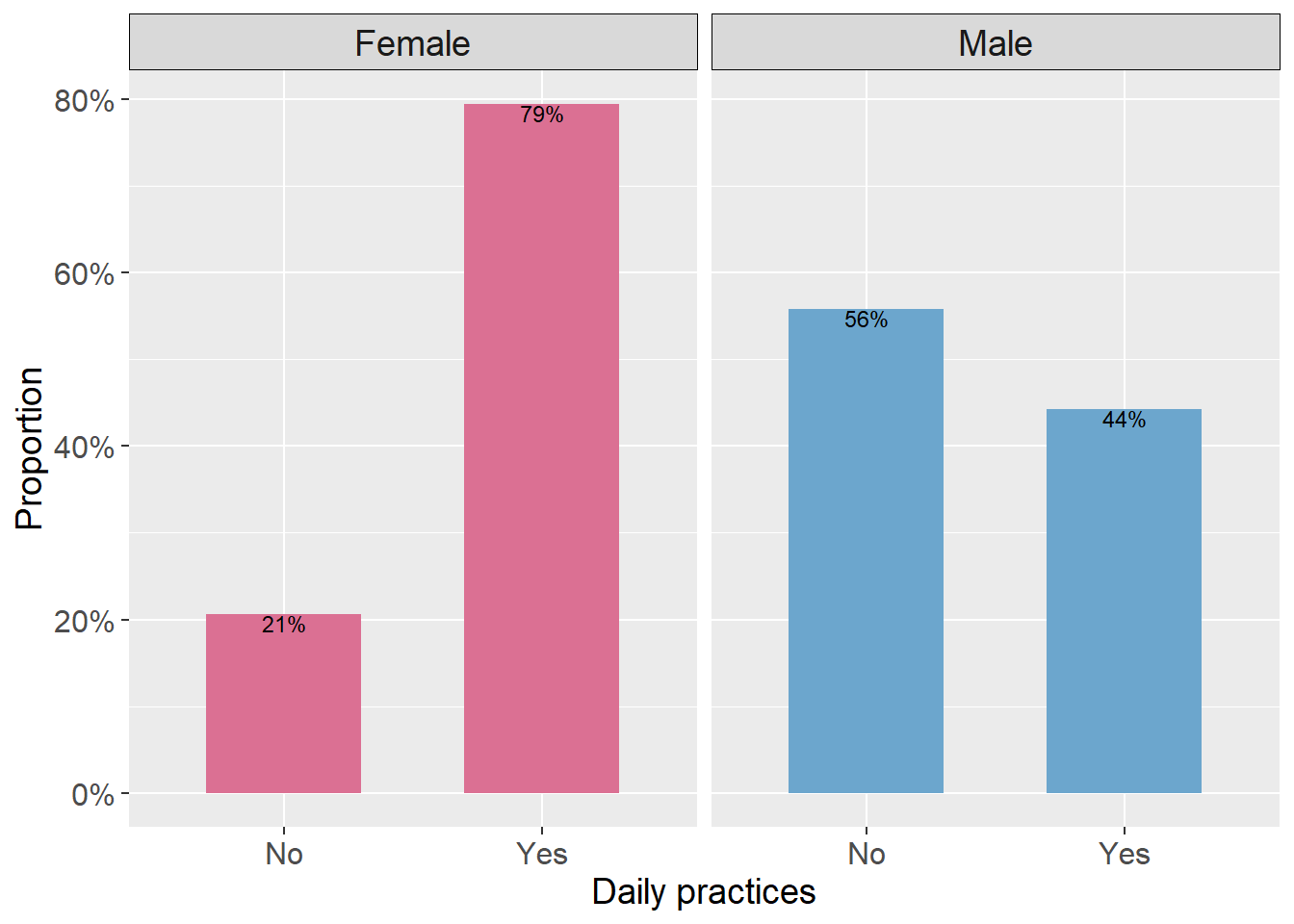
**

**b)**


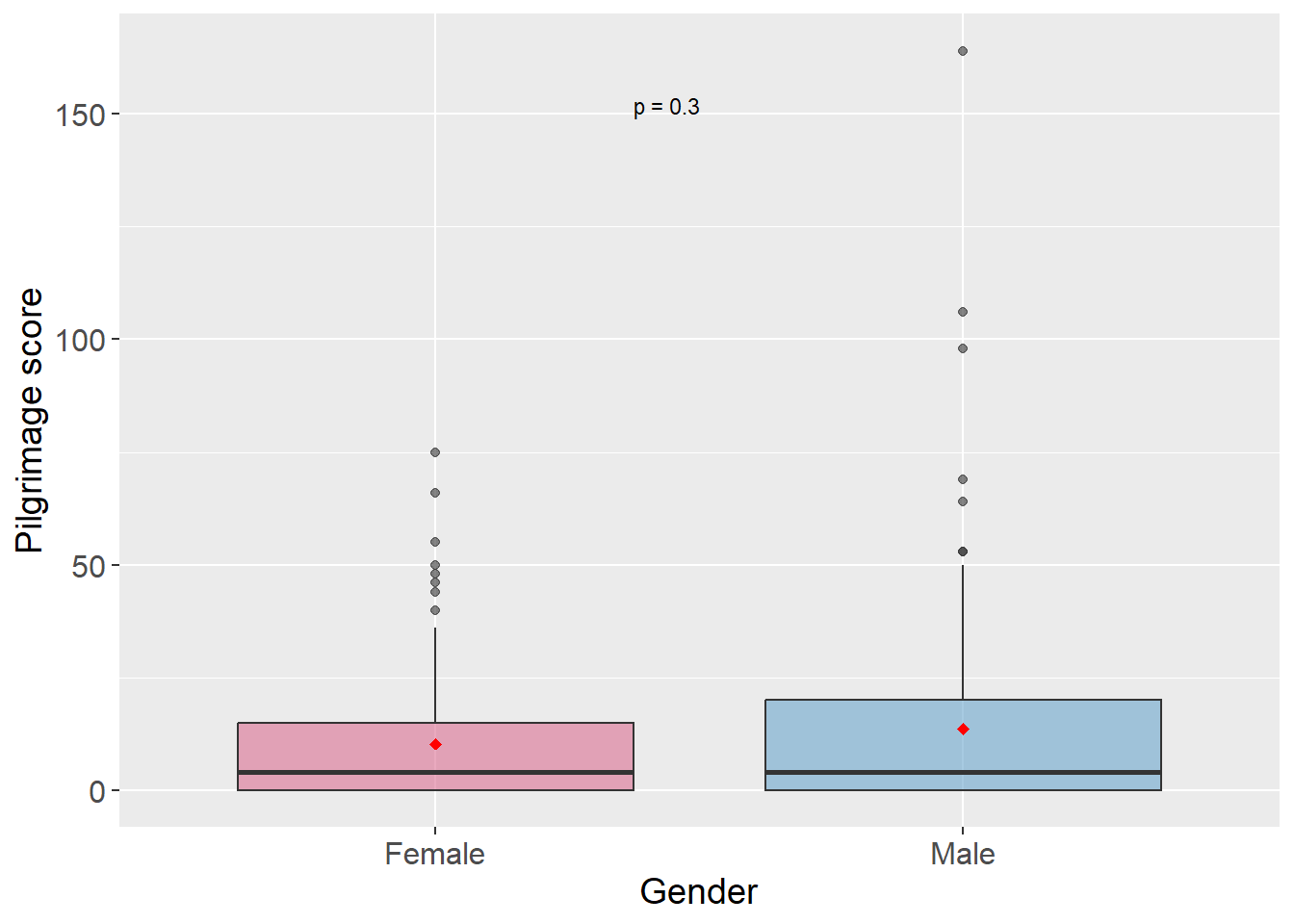


Figure S1. Distribution of religious variables classified by gender.

a) Daily practice; b) Pilgrimage score; *p* value was computed using Wilcoxon tests.


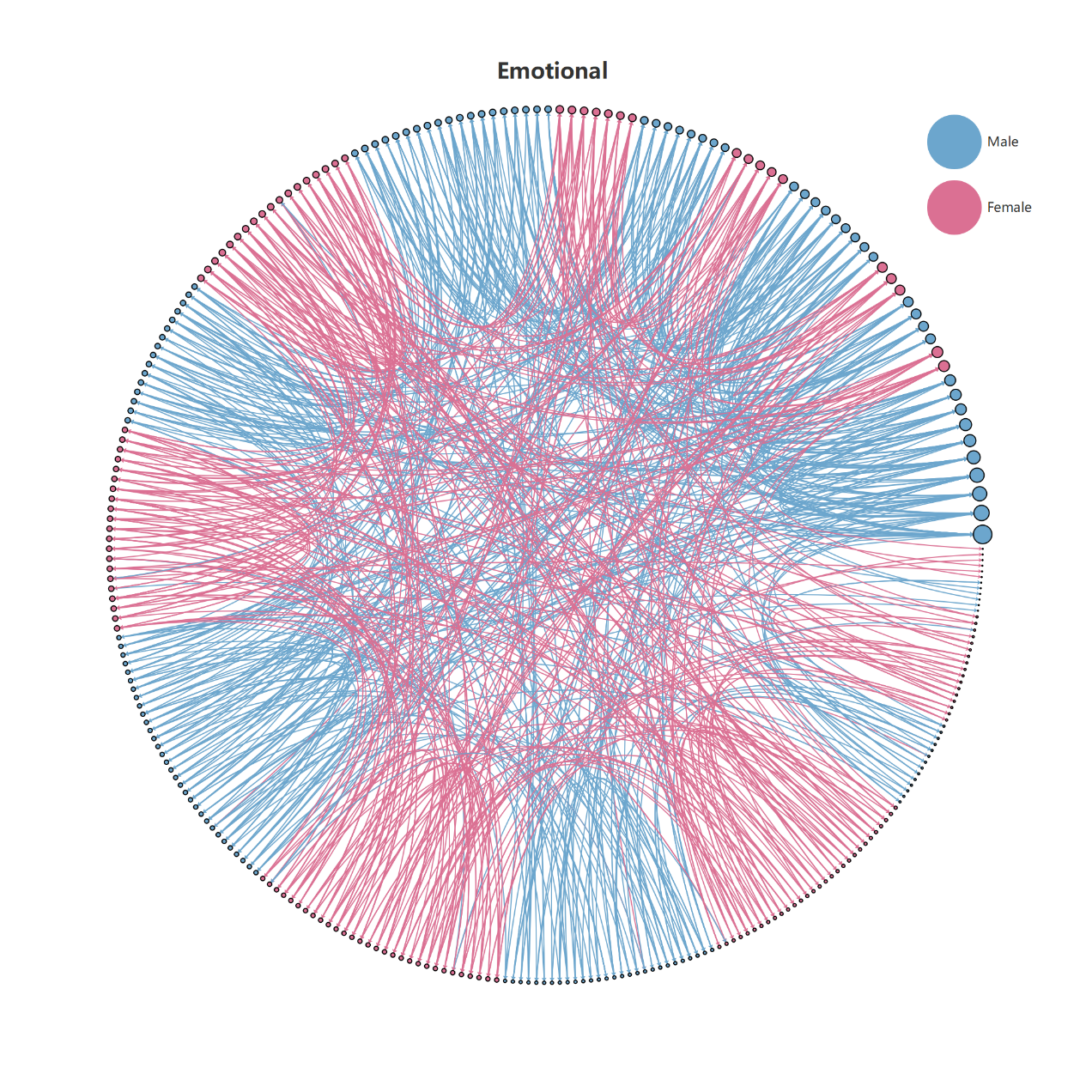


Figure S2. The emotional support network of the adult residents in the village. Nodes are coloured by gender and sized by in-degree value. Edges are directed, with an arrow directed from the person requesting support to the person providing it. Edges are coloured by the gender of alters. Nodes are ordered by the In-degree value.


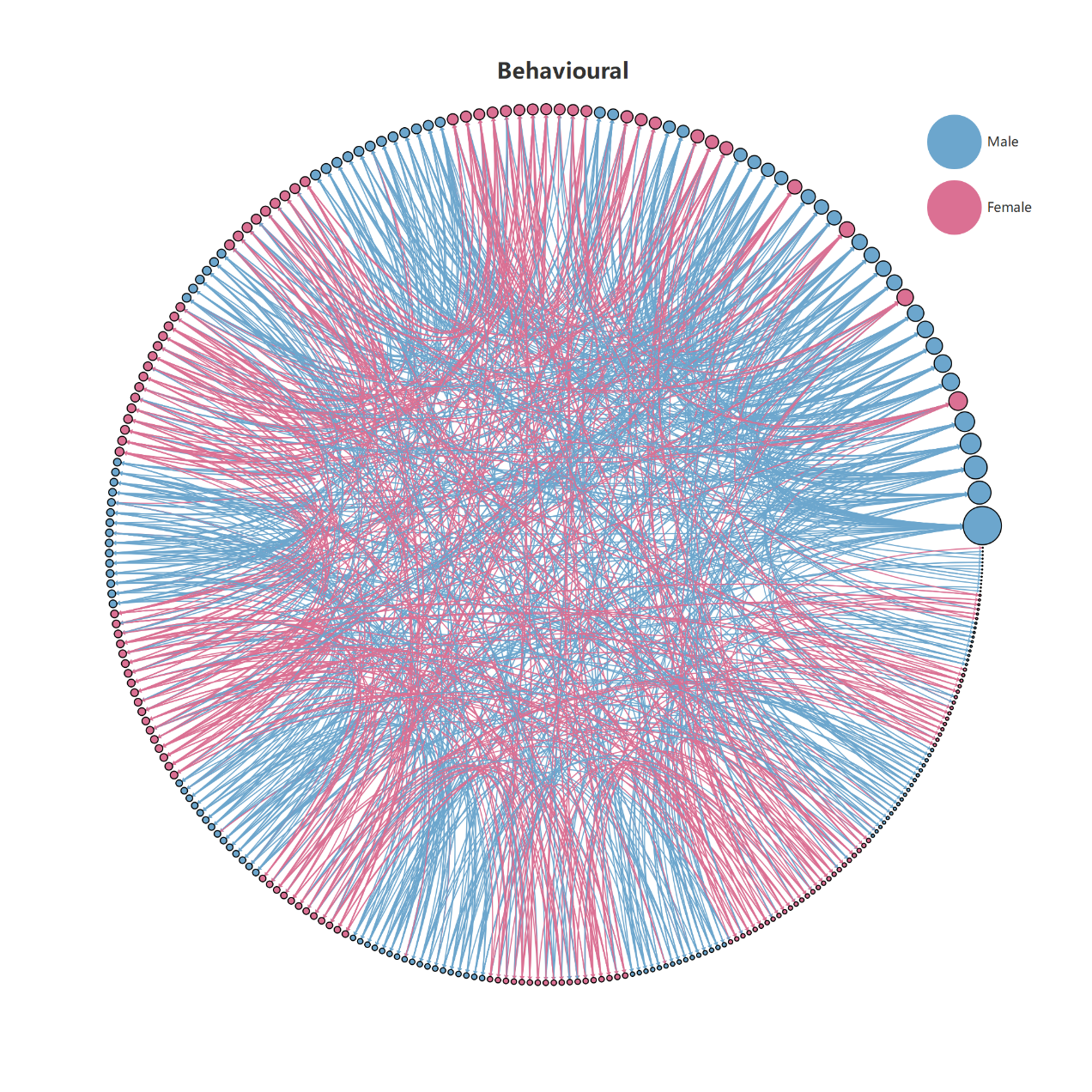


Figure S3. The behavioural support network of the adult residents in the village. Nodes are coloured by gender and sized by in-degree value. Edges are directed, with an arrow directed from the person requesting support to the person providing it. Edges are coloured by the gender of alters. Nodes are ordered by the In-degree value.


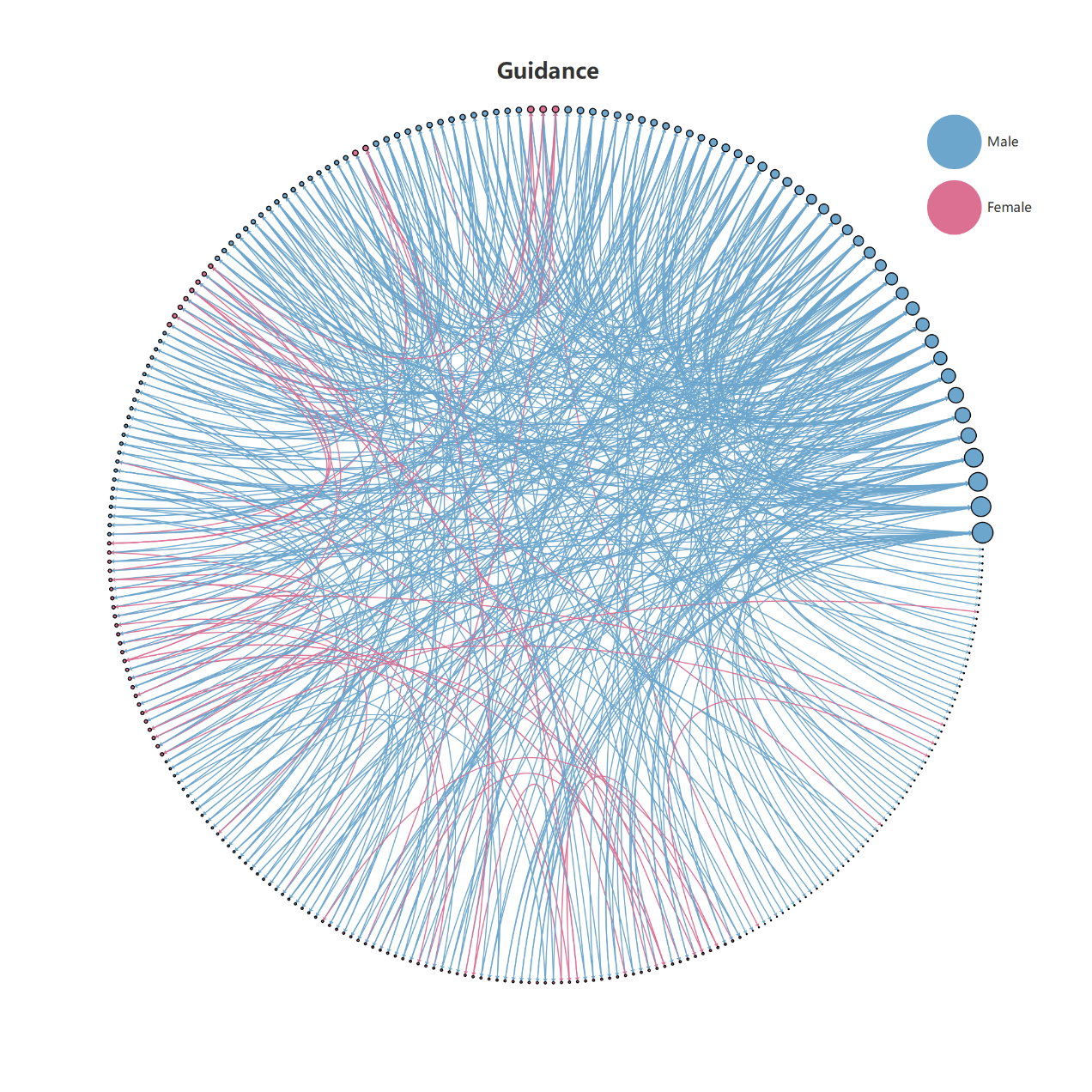


Figure S4. The guidance support network of the adult residents in the village. Nodes are coloured by gender and sized by in-degree value. Edges are directed, with an arrow directed from the person requesting support to the person providing it. Edges are coloured by the gender of alters. Nodes are ordered by the In-degree value.


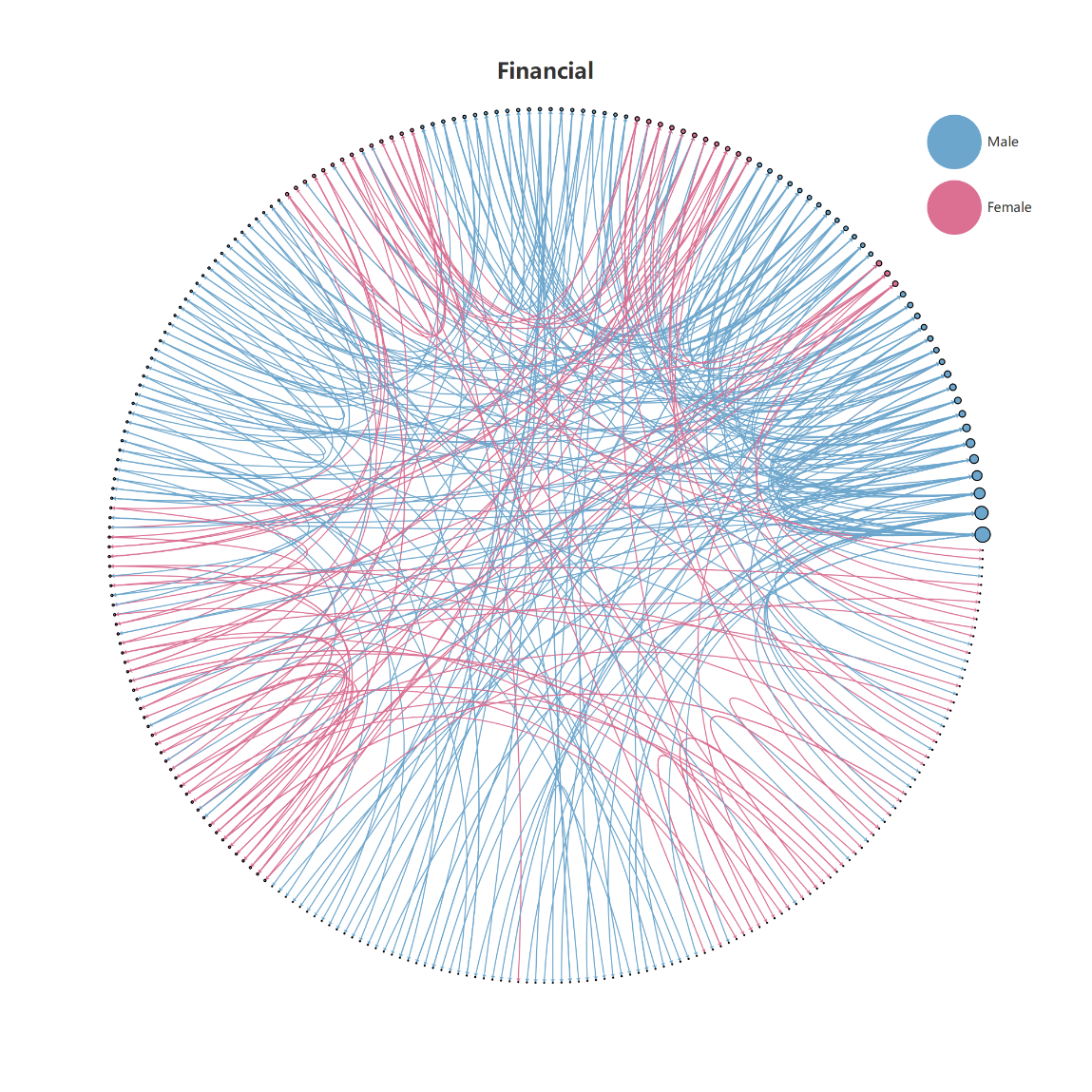


Figure S5. The financial support network of the adult residents in the village. Nodes are coloured by gender and sized by in-degree value. Edges are directed, with an arrow directed from the person requesting support to the person providing it. Edges are coloured by the gender of alters. Nodes are ordered by the In-degree value.


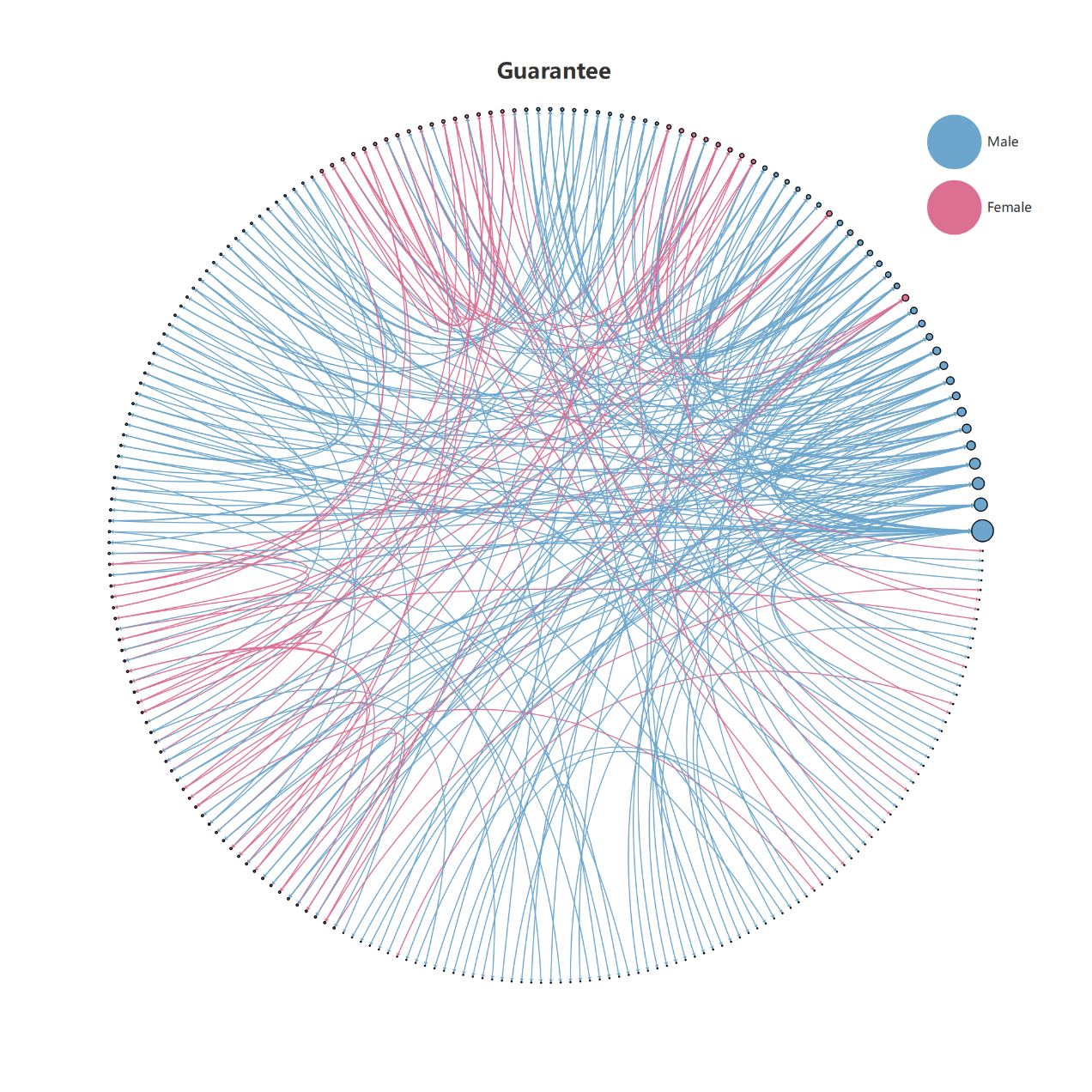


Figure S6. The guaranteed support network of the adult residents in the village. Nodes are coloured by gender and sized by in-degree value. Edges are directed, with an arrow directed from the person requesting support to the person providing it. Edges are coloured by the gender of alters. Nodes are ordered by the In-degree value


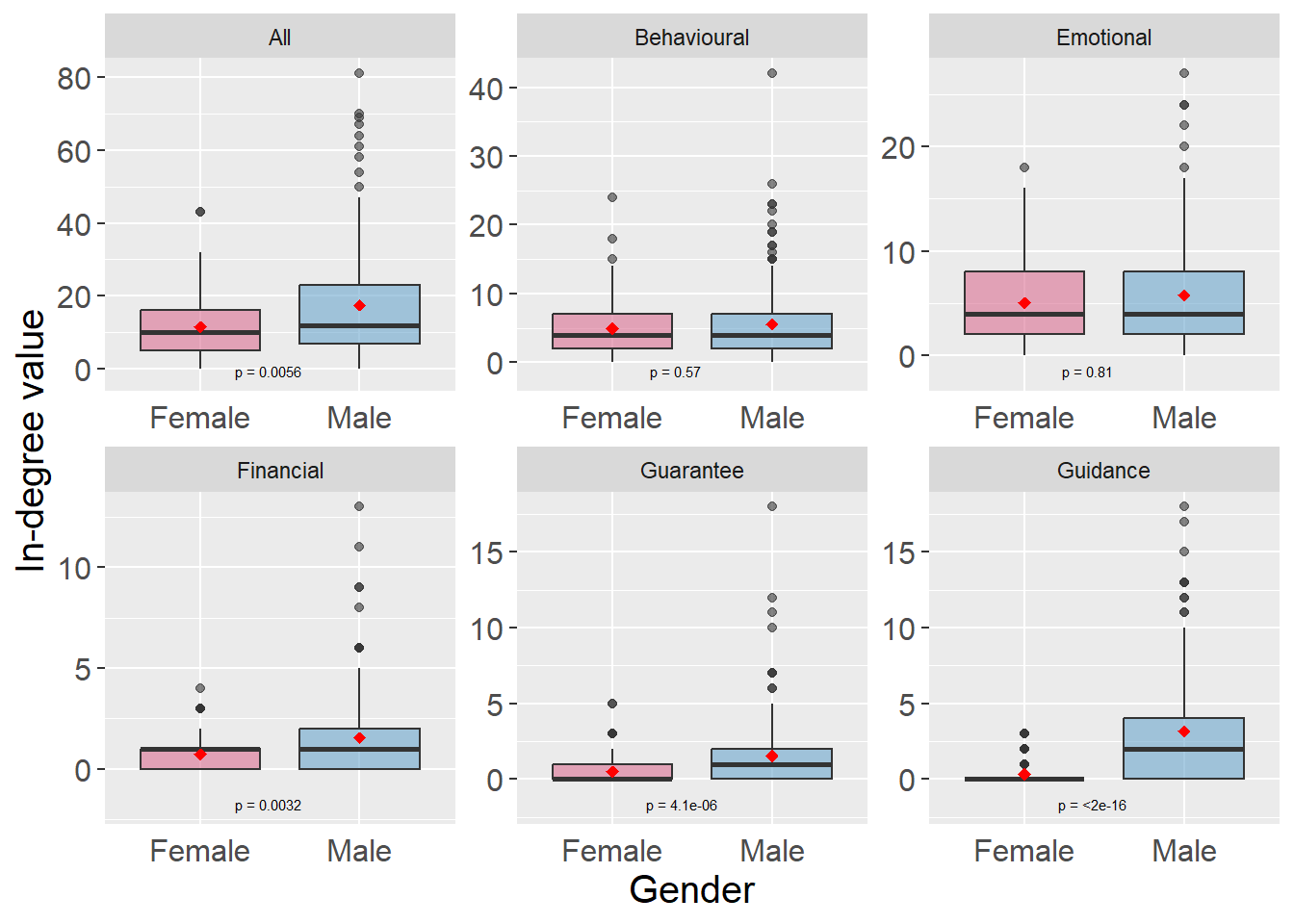


Figure S7. Gender-based distribution of in-degree values for the full personal network and each distinct supportive personal network, with Wilcoxon test results shown in each panel.


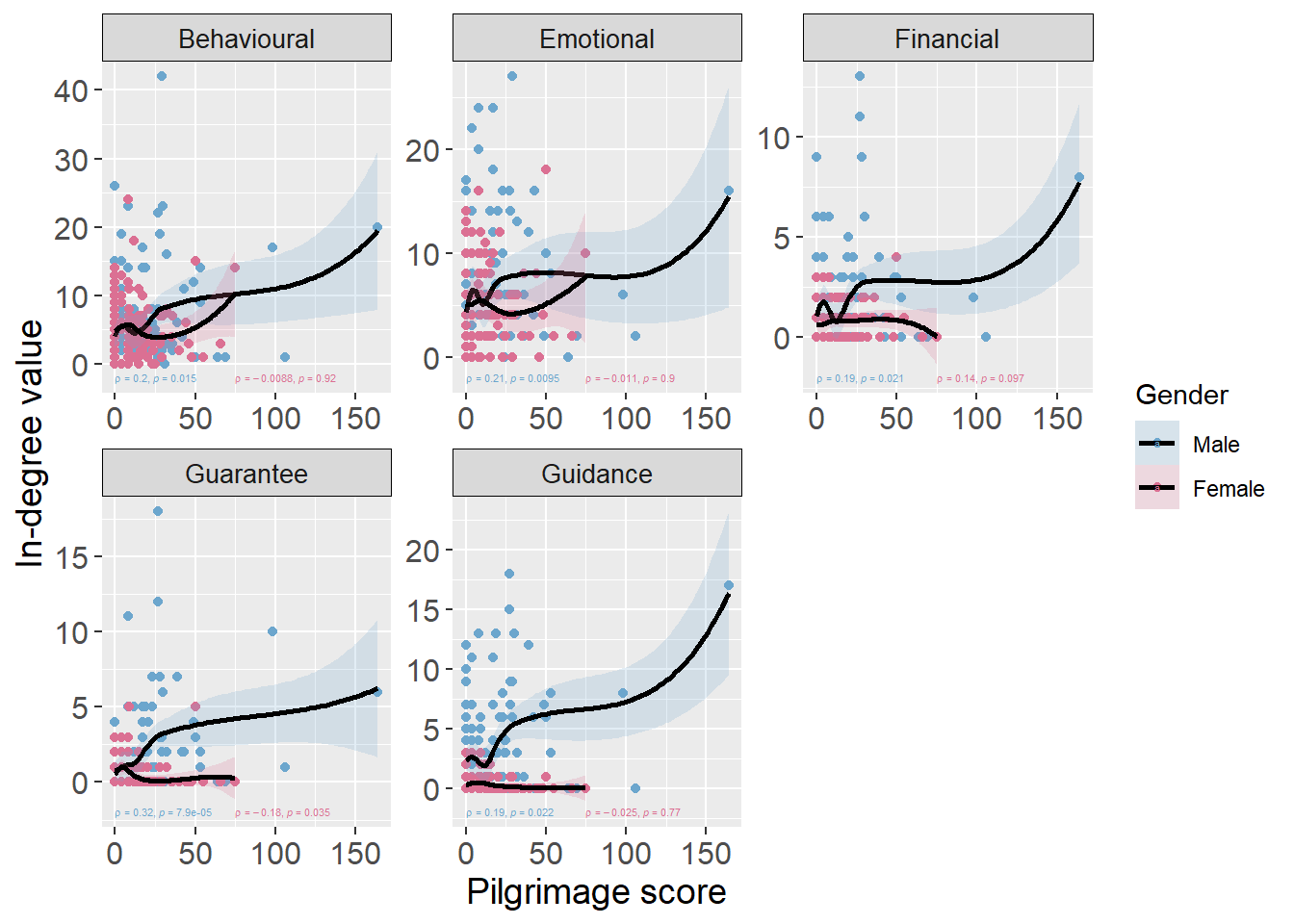


Figure S8. Correlation between pilgrimage and in-degree value in each specific personal network, with Spearman's rank correlation test results displayed in each panel. LOESS curves are illustrated by lines, with the shaded region denoting a 95% confidence interval. Females are depicted with red dots and bands, while males are represented by blue dots and bands. Spearman's correlation coefficients and associated *p*-values are colour-coded to match the corresponding gender.


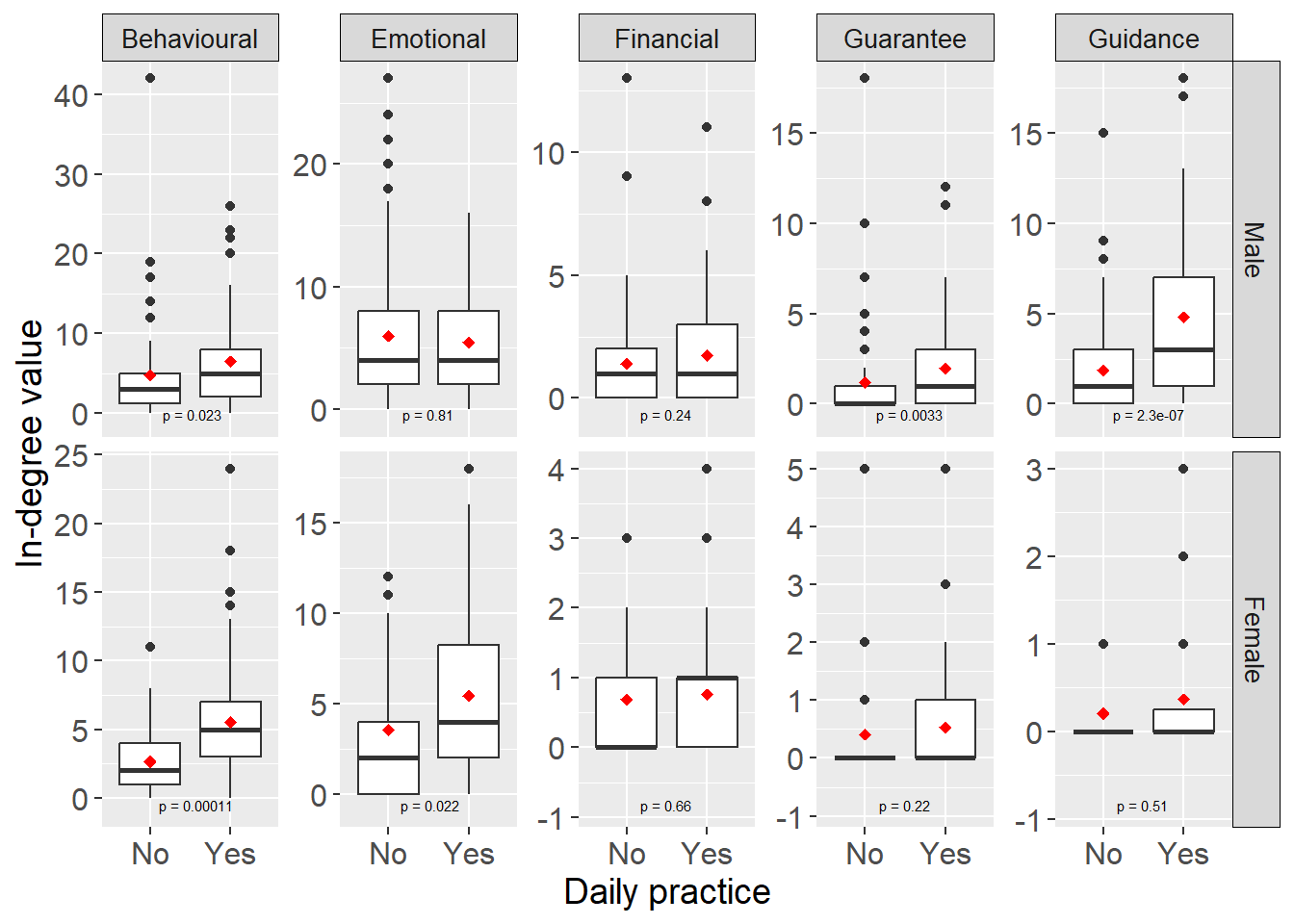


Figure S9. Correlation between daily religious practice and in-degree value in each specific personal networks, with Wilcoxon test results shown in each panel. Red diamonds denote the mean in-degree value. The box signifies the interquartile range (IQR); the central line indicates the median. Whiskers extend to 1.5 times the IQR; outliers are displayed as dots.


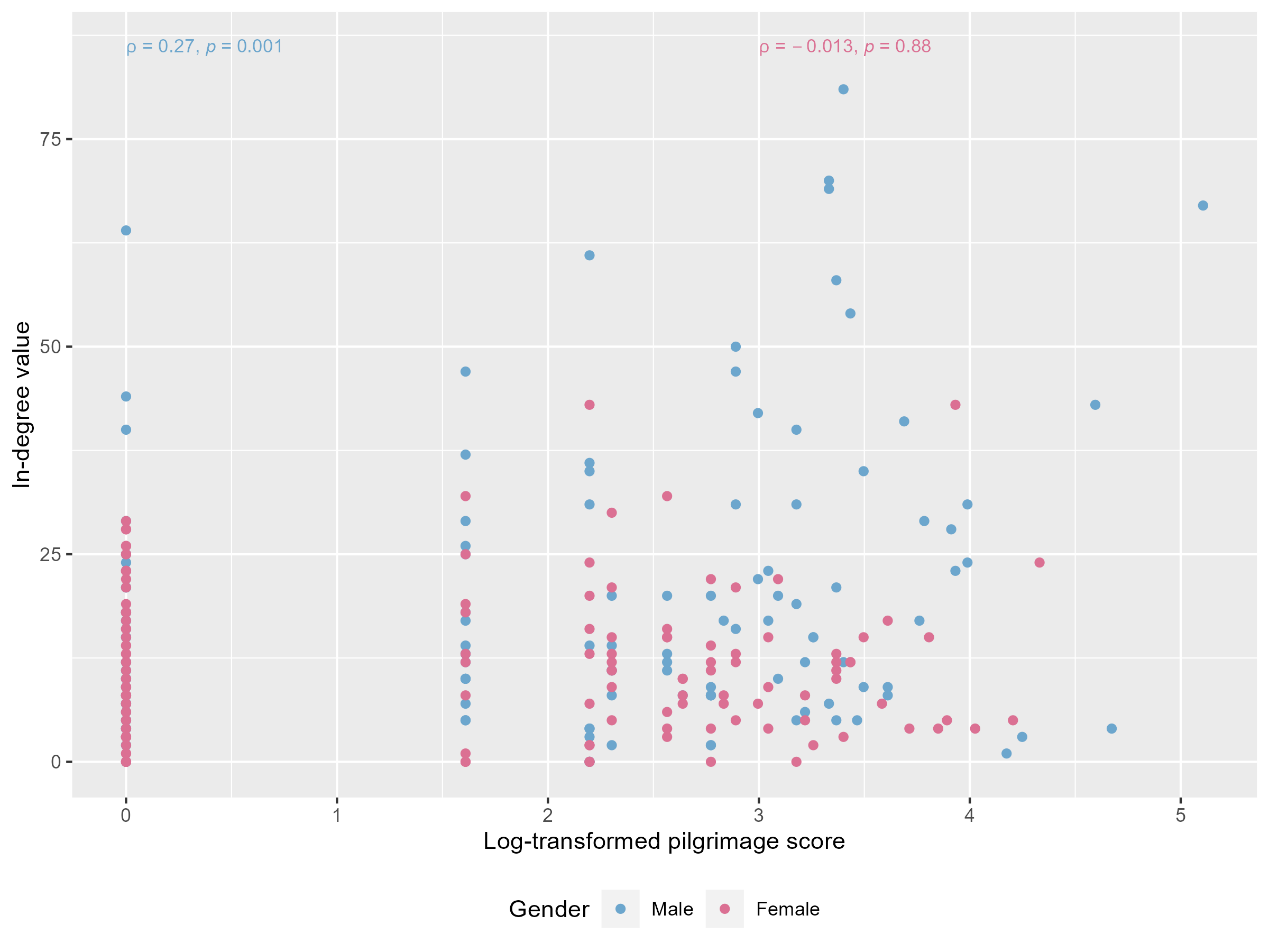
 a)


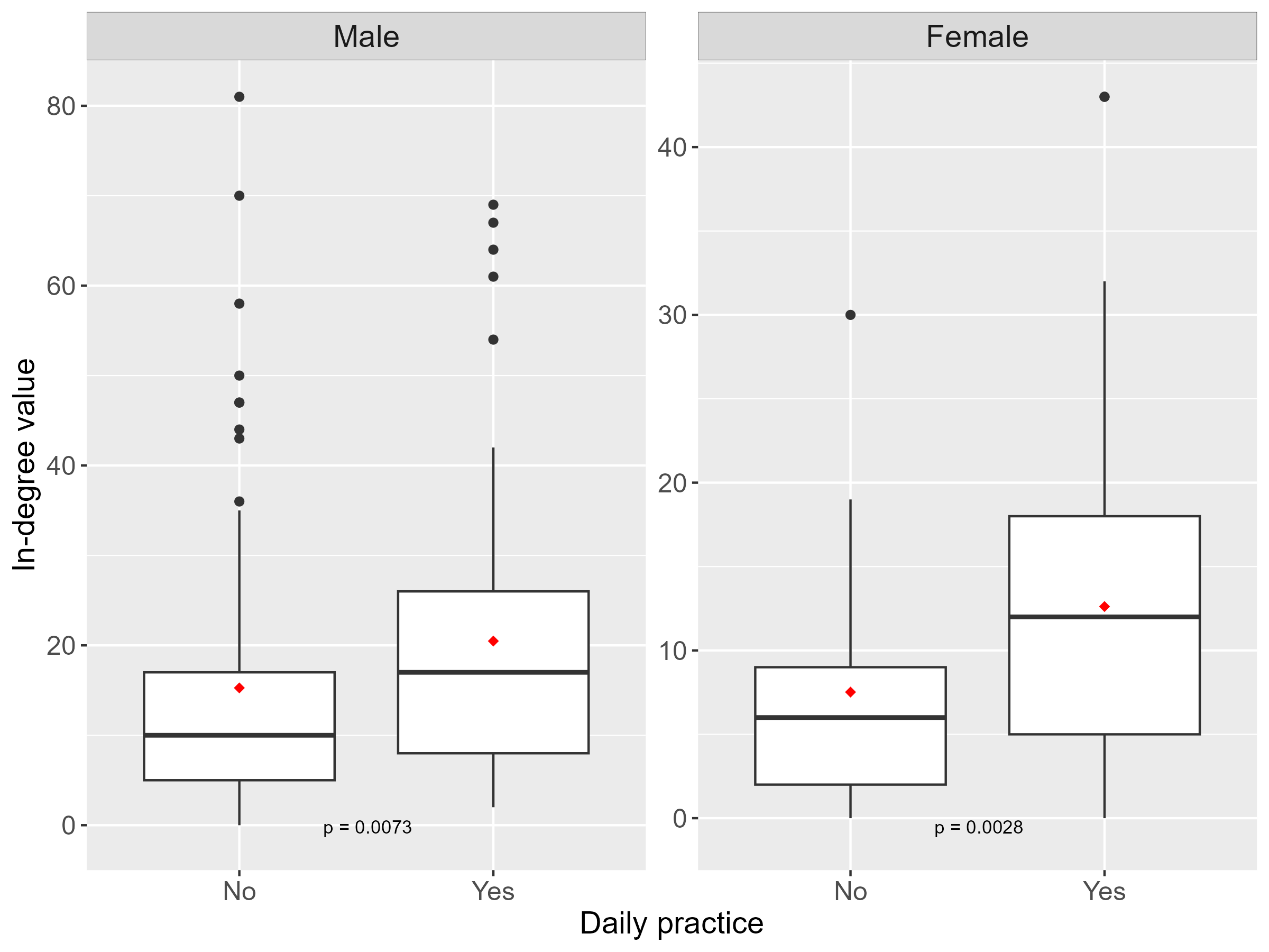
 b)

Figure S10. Correlation between religiosity and in-degree value in personal networks. a) The distribution of in-degree value varied by natural log-transformed pilgrimage score for both genders. Females are depicted with red dots and bands, while males are represented by blue dots and bands; Reported Spearman's correlation coefficients and associated *p* values are colour-coded to match the corresponding gender. b) The distribution of in-degree value varied by whether participating in daily religious practice regularly for both genders. Red diamonds denote the average in-degree value. The box signifies the interquartile range (IQR); the central line indicates the median. Whiskers extend to 1.5 times the IQR; outliers are displayed as dots. *p* values were computed using Wilcoxon tests and are reported within each panel.
